# A functional interplay of *hns* genes governs the control of the motile-to-biofilm lifestyle switch in *Burkholderia cenocepacia*

**DOI:** 10.1128/spectrum.00344-26

**Published:** 2026-09-02

**Authors:** Ana Júlia Virgílio Tonhetta, Ludie Andrea Chunga Mozombite, Nathalia dos Santos Oliveira, Isabella Carolina Rodrigues dos Santos Goes, Pedro Hadime Hori, Leo Kei Iwai, João Vitor de Lima Galhardo, Tie Koide, Marcelo Brocchi, Cristina Elisa Alvarez-Martinez, Rogerio Ferreira Lourenço

**Affiliations:** 1 Departamento de Genética, Evolução, Microbiologia e Imunologia, Instituto de Biologia, Universidade Estadual de Campinas 28132, Campinas, Brazil; 2 Departamento de Bioquímica e Imunologia, Faculdade de Medicina de Ribeirão Preto, Universidade de São Paulo 28133, Ribeirão Preto, Brazil; 3 Laboratório de Toxinologia Aplicada, Centro de Toxinas, Resposta-Imune e Sinalização Celular (LETA/CeTICS), Instituto Butantan 196591 , São Paulo, Brazil; 4 Departamento de Microbiologia, Imunologia e Parasitologia, Escola Paulista de Medicina, Universidade Federal de São Paulo 28105, São Paulo, Brazil; University of Manitoba, Winnipeg, Manitoba, Canada

**Keywords:** *Burkholderia*, gene expression regulation, virulence, nucleoid-associated protein, H-NS

## Abstract

The onset of the chronic stage of infection by bacteria in the *Burkholderia cepacia* complex depends on the controlled transition to the biofilm-associated lifestyle. In this study, we systematically investigated the effect of all five *hns* genes of *Burkholderia cenocepacia* H111 on the motile-to-biofilm lifestyle switch and pathogenicity. The *hns* genes are distributed across the three replicons (*hns*_1.1_ and *hns*_1.2_ on chromosome 1, *hns*_2_ on chromosome 2, and *hns*_3.1_ and *hns*_3.2_ on the megaplasmid pC3). We showed that deletion of either *hns*_1.1_ or *hns*_1.2_ affects cell growth and promotes the cell transition to the biofilm, with the major impact observed in cells lacking *hns*_1.2_. According to a comparative transcriptome analysis, the motile-to-biofilm switch linked to the absence of *hns*_1.2_ is supported by downregulation of flagellar and chemotaxis genes and upregulation of several genes involved in biofilm formation. In contrast, biofilm-related genes were downregulated; biofilm formation was reduced; and motility was increased in cells lacking *hns*_3.1_. An opposite impact of *hns*_1.2_ and *hns*_3.1_ on the motile-to-biofilm transition was also supported by using cells expressing these *hns* genes at high levels. Furthermore, *hns*_3.1_ and *hns*_1.1_ were shown to play a role in bacterial virulence in the *Galleria mellonella* infection model. Therefore, this study uncovers *hns* genes as new players of the motile-to-biofilm transition in *B. cenocepacia*.

**IMPORTANCE:**

Several opportunistic pathogenic bacteria can thrive as motile, free-living cells in soil and water, as well as in biofilm in their hosts. This includes the closely related species comprising the *Burkholderia cepacia* complex, which chronically colonize the airway in individuals with cystic fibrosis. Over the last two decades, the number of genes involved in the control of the motile-to-biofilm lifestyle switch has increased in this bacterial group, particularly in *Burkholderia cenocepacia*. In the present study, we uncover three *hns* genes that participate in the motile-to-biofilm lifestyle switch in *B. cenocepacia* H111 under the conditions tested. While two chromosomally encoded *hns* genes work to maintain cells at the motile stage, a megaplasmid-encoded *hns* gene facilitates the cell transition to biofilm. By systematically investigating all five *hns* genes, we considerably expand our understanding of the control of the motile-to-biofilm lifestyle switch in the *Burkholderia cepacia* complex.

## INTRODUCTION

Bacteria in the *Burkholderia cepacia* complex (Bcc) have been recognized as causative agents of bloodstream infections associated with contaminated solutions and other medical supplies (1–4). Additionally, these bacteria can establish a chronic pulmonary infection in individuals with cystic fibrosis (5). In this case, cells switch their lifestyle, developing into biofilm, which depends on the activation of specific transcriptional factors in response to increased intracellular concentration of c-di-GMP or autoinducers (6–13). These studies on the regulation of the motile-to-biofilm lifestyle switch have been particularly focused on *Burkholderia cenocepacia*, which is the Bcc species most commonly isolated from patients with cystic fibrosis (14).

In *Burkholderia cenocepacia* J2315, an H-NS protein (encoded by the BCAL0154 gene) was identified as an important factor for bacterial survival in the animal model *Caenorhabditis elegans*, and this effect on virulence was associated with the decreased expression of flagellar genes and reduced flagellum-dependent motility (15). The absence of BCAL0154 also enhanced biofilm formation (15). In another species of Bcc, namely, *Burkholderia multivorans*, a non-mucoid strain carrying an insertion sequence upstream of an *hns* gene, which corresponds to BCAL0122 in *B. cenocepacia* J2315, was isolated (16). This insertion sequence caused a fourfold increase in the expression of *hns*, leading to reduced bacterial adherence to bronchial epithelial cells and increased motility, in line with downregulation of genes involved in adhesion and upregulation of flagellar and chemotaxis genes (16). Therefore, these findings point to a critical role of H-NS proteins in the control of the motile-to-biofilm lifestyle switch in Bcc.

H-NS is a nucleoid-associated protein that participates in global gene expression regulation (17). This protein can both stiffen and bridge DNA, and both DNA-binding modes were proposed to lead to reduced gene expression (17). H-NS preferentially targets AT-rich DNA, rendering this protein a xenogeneic silencer (18, 19). Reduced expression of horizontally acquired DNA, which comprises several virulence genes, is critical for bacterial cells to thrive in their environmental niches, preventing a fitness cost associated with the expression of genes when they are not needed. However, bacteria have evolved mechanisms to relieve genes from the H-NS-mediated silencing, such as (i) conformational changes of H-NS driven by direct sensing of physical and chemical clues (20–22), (ii) H-NS dissociation from DNA promoted by transcriptional factors that are induced and/or activated under specific conditions and bind DNA with higher affinity than H-NS does (23), and (iii) changes in the acetylated or phosphorylated pool of H-NS molecules in the presence of environmental or nutritional signals (24–27). Therefore, H-NS enables cells to regulate gene expression in response to external signals, including those encountered by bacteria while colonizing their hosts.

Notably, the abovementioned studies were not designed to systematically investigate H-NS proteins in species belonging to Bcc. Rather, strains in which a mobile DNA element was inserted into or near an *hns* gene were isolated while screening colonies for new virulence factors. Here, we determined the relative contribution of all five *hns* genes to the physiology and pathogenicity of *Burkholderia cenocepacia* H111, including the genes homologous to those analyzed in *B. cenocepacia* J2315 and *B. multivorans*. We showed that the two *hns* genes on chromosome 1 are important for the control of the motile-to-biofilm switch. Additionally, these genes are hierarchically organized in a regulatory cascade, with one gene positioned at the top, silencing the second gene and affecting several biological processes in the cell. In contrast, the absence of one of the two *hns* genes on the megaplasmid pC3 showed opposite phenotypes, suggesting this gene may act as a negative regulator of the master *hns* gene in the bacterium. Therefore, our data imply a functional interplay of *hns* genes controlling the motile-to-biofilm transition in *B. cenocepacia*.

## RESULTS

### H-NS proteins are produced in *B. cenocepacia* growing in a rich medium

**Fig 1:**
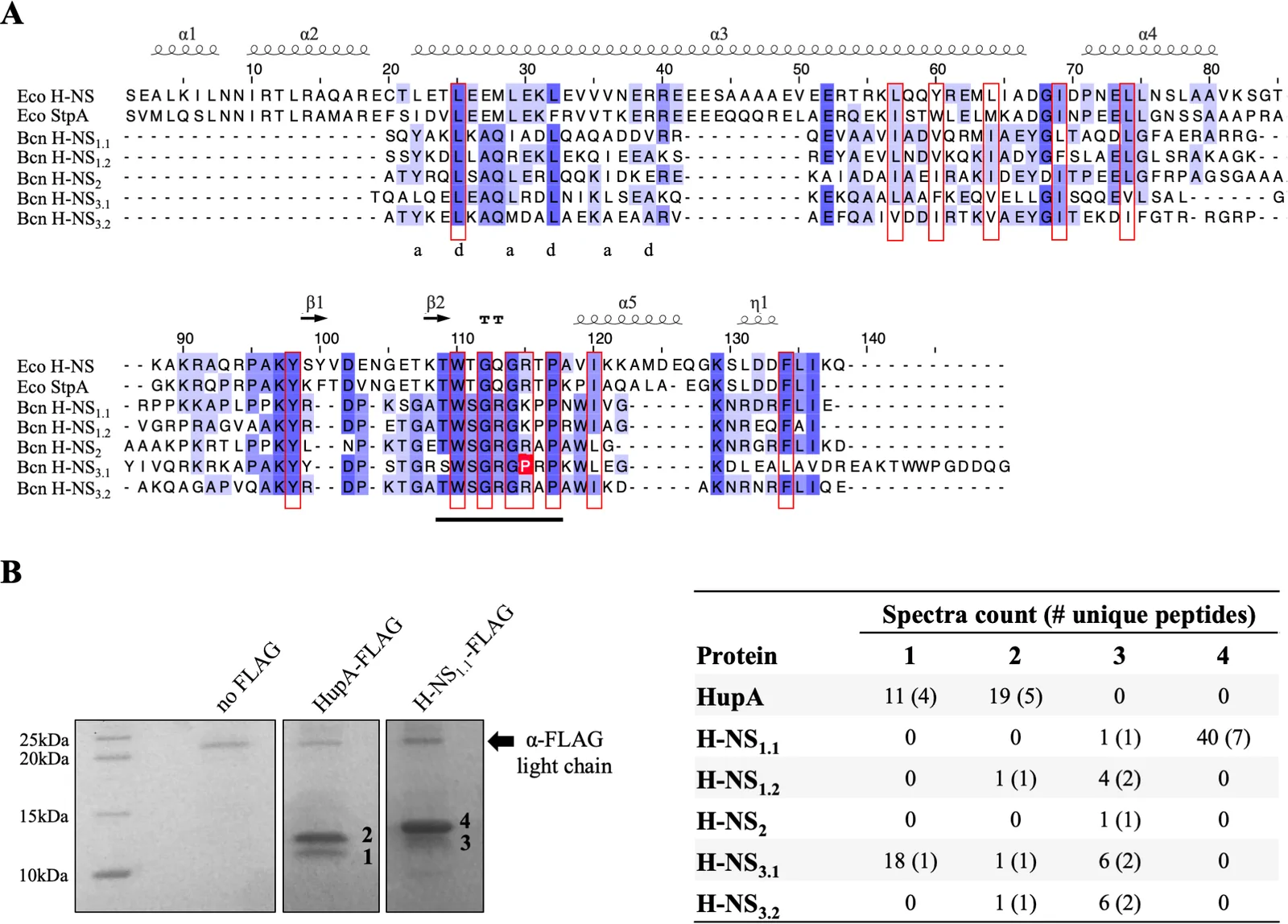
*In silico* analysis of H-NS proteins and their detection in *B. cenocepacia* H111. (**A**) Alignment of the deduced amino acid sequences of H-NS homologs in *E. coli* K-12 and *B. cenocepacia* H111. For *E. coli* H-NS, helices α1–α4 comprise the oligomerization domain, with the dimerization sites 1 and 2 encompassing residues 1–41 and 56–80, respectively. β1–η1 refers to the DNA-binding domain, and the linker corresponds to the region between the two protein domains. Conserved residues previously shown to contribute to gene silencing are marked as red rectangles. The “a” and “d” positions of the predicted coiled-coil heptad repeats are indicated below the alignment. The AT-hook motif is denoted by a black bar below the sequence, and a proline residue at the AT-hook sequence of H-NS_3.1_ is highlighted in red. (**B**) Identification of proteins co-purified with HupA and H-NS_1.1_. Saturated cultures of cells expressing either HupA-FLAG or H-NS_1.1_-FLAG were diluted in fresh medium and grown to exponential phase (OD_600nm_ ≃ 0.5). The FLAG-tagged proteins were immunoprecipitated using anti-FLAG affinity gel. The eluates were resolved by SDS-PAGE, and four protein bands were isolated (denoted as 1–4 in the left panel). Peptides obtained from in-gel digestion of proteins with trypsin were analyzed by LC-MS/MS (right panel). Parental cells, expressing both proteins without the FLAG tag (no FLAG in the left panel), were used as control. The complete list of proteins identified from each band is shown in Table S1.

For simplicity, we named the H-NS-encoding genes according to their positions on the three replicons of *B. cenocepacia* H111, as follows: *hns*_1.1_ (I35_RS00650, BCAL0122) and *hns*_1.2_ (I35_RS00810, BCAL0154) on chromosome 1, *hns*_2_ (I35_RS29410, BCAM2711) on chromosome 2, and *hns*_3.1_ (I35_RS30600, BCAS0118) and *hns*_3.2_ (I35_RS31030, BCAS0190) on the megaplasmid pC3. By analyzing the amino acid sequences deduced from these *hns* genes and comparing them with H-NS and its homolog StpA in *Escherichia coli* K-12, we observed that (i) H-NS proteins in *B. cenocepacia* are shorter than *E. coli* H-NS and StpA, remarkably in the region corresponding to the oligomerization domain (positions 1–80 in the alignment); (ii) *B. cenocepacia* H-NS proteins may have up to three consecutive heptad repeats in their N-termini (positions 22–41 in the alignment), which could be involved in self-associating subunits into a coiled-coil structure; (iii) The C-terminus is the most conserved region of *B. cenocepacia* H-NS proteins, especially at the AT-hook motif (positions 109–117 in the alignment) putatively involved in the direct association with DNA; (iv) the linker encompasses positively charged residues (positions 81–94 in the alignment), which may be important for the DNA-binding activity; and (v) several amino acid residues that are critical for gene silencing by *E. coli* H-NS (28) are conserved in *B. cenocepacia* H-NS proteins (highlighted by red rectangles in Fig. 1A). Notably, H-NS_3.1_ possesses a short C-terminal extension and a proline in place of a positively charged residue at the AT-hook motif, in a position expected to be critical for DNA binding (Fig. 1A). Therefore, our *in silico* analysis provides evidence that the *B. cenocepacia* replicons encode functional H-NS proteins, but H-NS_3.1_ may deviate from what can be considered a canonical H-NS protein with respect to the DNA-binding properties.

With the aim of investigating whether the H-NS proteins are produced in *B. cenocepacia* H111 growing in a rich medium, we sought to purify nucleoprotein complexes from nucleoid fragmentation and identify proteins by mass spectrometry. We created a merodiploid strain by integrating the *hupA*-FLAG-containing pGPI-SceI vector into the *hupA* native locus on chromosome 1 (Fig. S1A). HupA was chosen due to the high abundance of its orthologs in the nucleoid of different bacteria (29). Consistent with the expected ability of HupA to self-associate (30, 31), two protein bands were observed upon analyzing the eluate of a co-immunoprecipitation (co-IP) experiment carried out with the merodiploid strain (Fig. 1B). While Western blotting detected a single band (Fig. S1B), which may correspond to the FLAG-tagged protein, mass spectrometry indicated the presence of HupA in both bands (Fig. 1B and Table S1). Therefore, both tagged and untagged proteins were purified. Using mass spectrometry, we also identified three H-NS proteins, namely, H-NS_1.2_, H-NS_3.1_, and H-NS_3.2_, in the slower-migrating band and H-NS_3.1_ in the faster-migrating band (Fig. 1B and Table S1). In addition, we detected a smear that disappeared when the eluate was first treated with a nuclease (Fig. S1C), suggesting co-purification of nucleoid fragments, as previously observed for other nucleoid-associated proteins (32). Therefore, this experimental approach led to the purification of components expected to be part of the bacterial nucleoid, including H-NS proteins.

We reasoned that H-NS proteins could be further enriched in a co-IP experiment using an H-NS-FLAG-producing strain, as H-NS homologs are expected to form heterooligomers. To accomplish this, another merodiploid strain was generated by inserting *hns*_1.1_-FLAG-carrying pGPI-SceI into the *hns*_1.1_ native locus (Fig. S1A). However, a FLAG-tagged protein was not recovered during co-IP (Fig. S1B), suggesting low expression levels of *hns*_1.1_ from its own promoter. On the other hand, using a strain harboring *hns*_1.1_-FLAG under the control of a rhamnose-inducible promoter in a replicative plasmid, we were able to detect a FLAG-tagged protein by Western blotting (Fig. S1B) and two protein bands in a denaturing gel (Fig. 1B). H-NS_1.1_ was identified with no other H-NS protein in the slower-migrating band, but all five predicted H-NS proteins were found in the faster-migrating band (Fig. 1B and Table S1). Strikingly, not only H-NS_1.1_ and H-NS_2_ could be identified using the H-NS_1.1_-FLAG-producing strain but also the other three H-NS proteins seemed to be more abundant in the co-IP eluate using this strain relative to the experiment with HupA-FLAG-producing cells (Fig. 1B and Table S1). Taken together, our data demonstrate that *hns* genes are expressed in *B. cenocepacia* H111 under the growth conditions tested.

### *hns* genes play critical roles in *B. cenocepacia* physiology and pathogenicity

**Fig 2:**
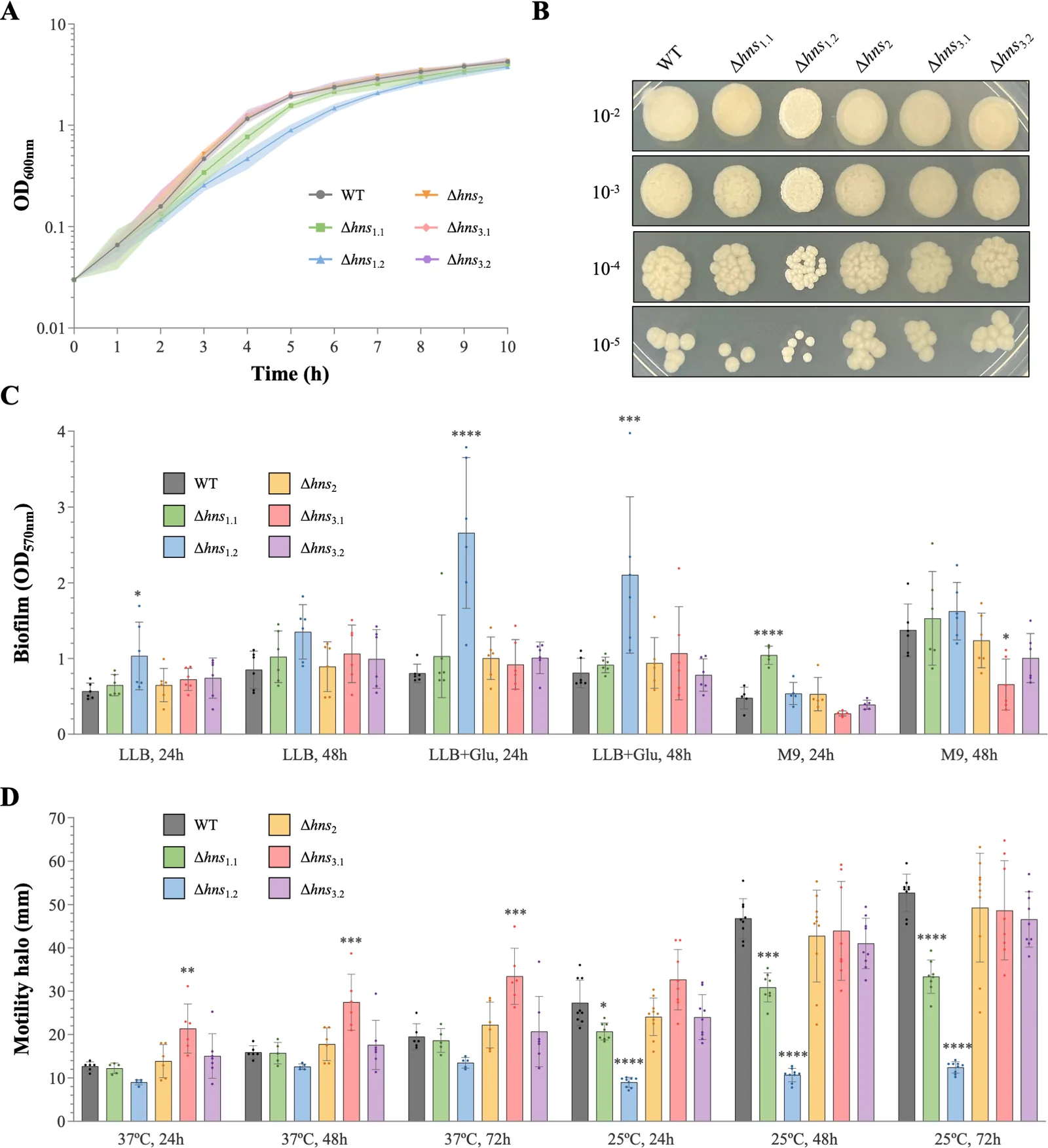
Both *hns* genes on chromosome 1 are important for bacterial growth and the motile lifestyle, while one of the *hns* genes on the megaplasmid pC3 contributes to biofilm formation. (**A**) Growth curves of the *hns* mutants and parental cells. Saturated cultures were diluted in fresh LLB medium, and the absorbance at 600 nm (OD_600nm_) was measured at the indicated time points. Data are mean values ± standard deviations of three independent experiments. (**B**) Colony morphology of the *hns* mutants and parental cells. Saturated cultures were diluted in fresh LLB medium, grown to exponential phase (OD_600nm_ ≃ 0.5), serially diluted, and spotted on an LLB agar plate. The plate was imaged after 72 h at 25°C. (**C**) Biofilm formation of the *hns* mutants and parental cells. Saturated cultures were diluted in fresh media (LLB, LLB supplemented with 0.5% glucose, and M9) and grown in microtiter polystyrene dishes under static conditions. At the indicated time points, attached cells were stained with 1% crystal violet, and OD_570nm_ was determined. Data are mean values ± standard deviations of at least two independent experiments, with two to three technical replicates each. (**D**) Swarming motility of the *hns* mutants and parental cells. Saturated cultures were diluted in fresh LLB medium, grown to OD_600nm_ = 0.4, and spotted on semisolid plates. The diameter of the motility halo was determined at the indicated time points, and data are mean values ± standard deviations of at least three independent experiments with two to three technical replicates each. Statistical analysis was carried out using one-way ANOVA followed by Dunnett’s test to compare the mutant strains to a single control (WT) (*, *P* < 0.05; **, *P* < 0.01; ***, *P* < 0.001; ****, *P* < 0.0001).

To determine the relative contribution of the *hns* genes to *B. cenocepacia* physiology and pathogenicity, we individually deleted each gene by two homologous recombination events. Interestingly, two different populations with respect to colony size were found while screening for *hns*_1.2_ deletion. All colonies that appeared within 2–3 days reverted to the parental genotype, while colonies observed after 5–7 days were identified as Δ*hns*_1.2_ mutants. This delayed growth relative to parental cells was also demonstrated in liquid complex medium (Fig. 2A). On the other hand, no difference in colony size was observed during isolation of the other four *hns* mutant strains. When these mutants were evaluated in liquid complex medium, a growth defect was observed for the *hns*_1.1_ deletion mutant (Fig. 2A). Even though the Δ*hns*_1.1_ and Δ*hns*_1.2_ strains reached similar OD_600nm_ at the end of a 10 h growth experiment, doubling times estimated for these mutant strains at the beginning of the growth curves were significantly higher when compared to that of parental cells (Fig. S2A). Therefore, these results indicate that *hns*_1.1_ and *hns*_1.2_ are both important for *B. cenocepacia* growth.

Strikingly, colonies of the Δ*hns*_1.2_ strain showed a whitish appearance in complex medium (Fig. 2B) and developed a ring in a glass test tube at the air-liquid interface when grown in the same medium under shaking conditions (Fig. S2), suggesting significant changes in the cell surface that enable this mutant strain to adhere more efficiently to an abiotic surface. These phenotypes prompted us to quantify biofilm mass in static cultures growing in polystyrene microtiter plates. When grown in complex medium, cells deleted for *hns*_1.2_ showed increased adherence at the air-liquid interface with respect to parental cells, and the impact of *hns*_1.2_ deletion on this phenotype was even more pronounced when the medium was supplemented with glucose (Fig. 2C). Notably, we observed aggregates at the bottom of the wells, which are easily lost during biofilm staining, in cultures of the mutant strain grown in the absence of glucose (Fig. S2C). We also compared the strains in minimal medium but found no altered biofilm formation in the Δ*hns*_1.2_ strain (Fig. 2C). On the other hand, the mutants Δ*hns*_1.1_ and Δ*hns*_3.1_ showed increased and decreased biofilm, respectively, relative to the parental strain in minimal medium (Fig. 2C). Therefore, using different media, we showed that *hns*_1.1_ and *hns*_1.2_ negatively affect biofilm formation, while *hns*_3.1_ promotes this phenotype in *B. cenocepacia* H111.

We also investigated the effect of deleting the *hns* genes on the swarming motility. At 37°C, the motility halo was increased in the *hns*_3.1_ deletion mutant when compared to the parental strain (Fig. 2D; Fig. S2D). By reducing the temperature to 22°C–25°C, we observed (i) an increase in swarming motility of the wild-type cells; (ii) an important role of both *hns*_1.1_ and *hns*_1.2_ in motility, since cells lacking these genes were found to be defective in spreading on a semisolid surface; and (iii) no altered swarming motility of the *hns*_3.1_ mutant relative to the parental strain (Fig. 2D; Fig. S2D). Together, the data imply that *hns*_1.1_ and *hns*_1.2_ support swarming motility, whereas an opposite role can be linked to *hns*_3.1_.

**Fig 3:**
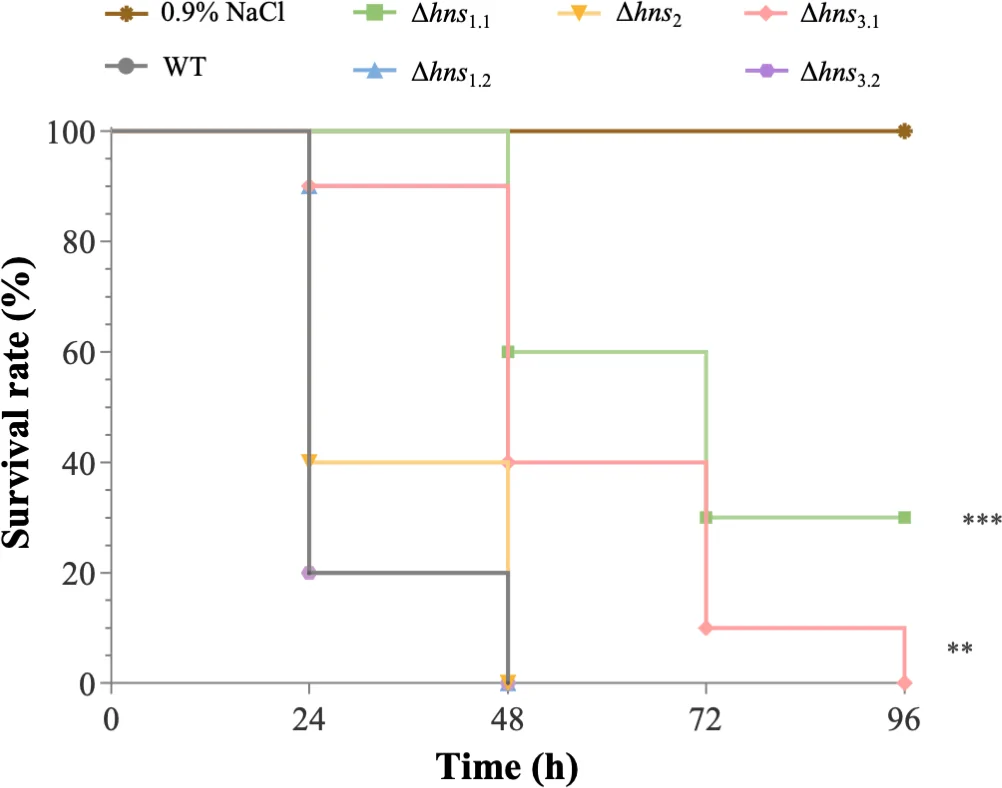
Two *hns* genes play a role in the virulence of *B. cenocepacia* H111. *G. mellonella* at the larval stage was infected with the *hns* mutants and parental cells grown to exponential phase (OD_600nm_ ≃ 0.5) and diluted to approximately 10^7^ CFU/mL. The 10 μL aliquots injected in each larva were estimated as containing 8.6 × 10^5^ (WT), 1.4 × 10^5^ (Δ*hns*_1.1_), 2.6 × 10^5^ (Δ*hns*_1.2_), 8.0 × 10^5^ (Δ*hns*_2_), 1.1 × 10^6^ (Δ*hns*_3.1_), and 1.8 × 10^6^ (Δ*hns*_3.2_). The Kaplan-Meier survival curves were analyzed using a log-rank (Mantel-Cox) test (**, *P* < 0.01; ***, *P* < 0.001). Results from additional experiments are shown in Fig. S3.

*Galleria mellonella* has been exploited as an animal model to study the pathogenicity of *B. cenocepacia* (33). Therefore, we compared parental cells and the *hns* mutants with respect to their ability to kill *G. mellonella* and observed that the *hns*_1.1_ deletion mutant shows a reduced virulence phenotype when compared to the wild-type strain (Fig. 3). In addition, the Δ*hns*_3.1_ strain plays a role in the virulence of *B. cenocepacia* H111, showing a delay in the killing kinetics when compared to wild-type cells (Fig. 3). Thus, the data may indicate a relationship between *hns* genes and virulence in *B. cenocepacia* H111.

### *hns*_1.2_ controls the expression of several virulence genes

**Fig 4:**
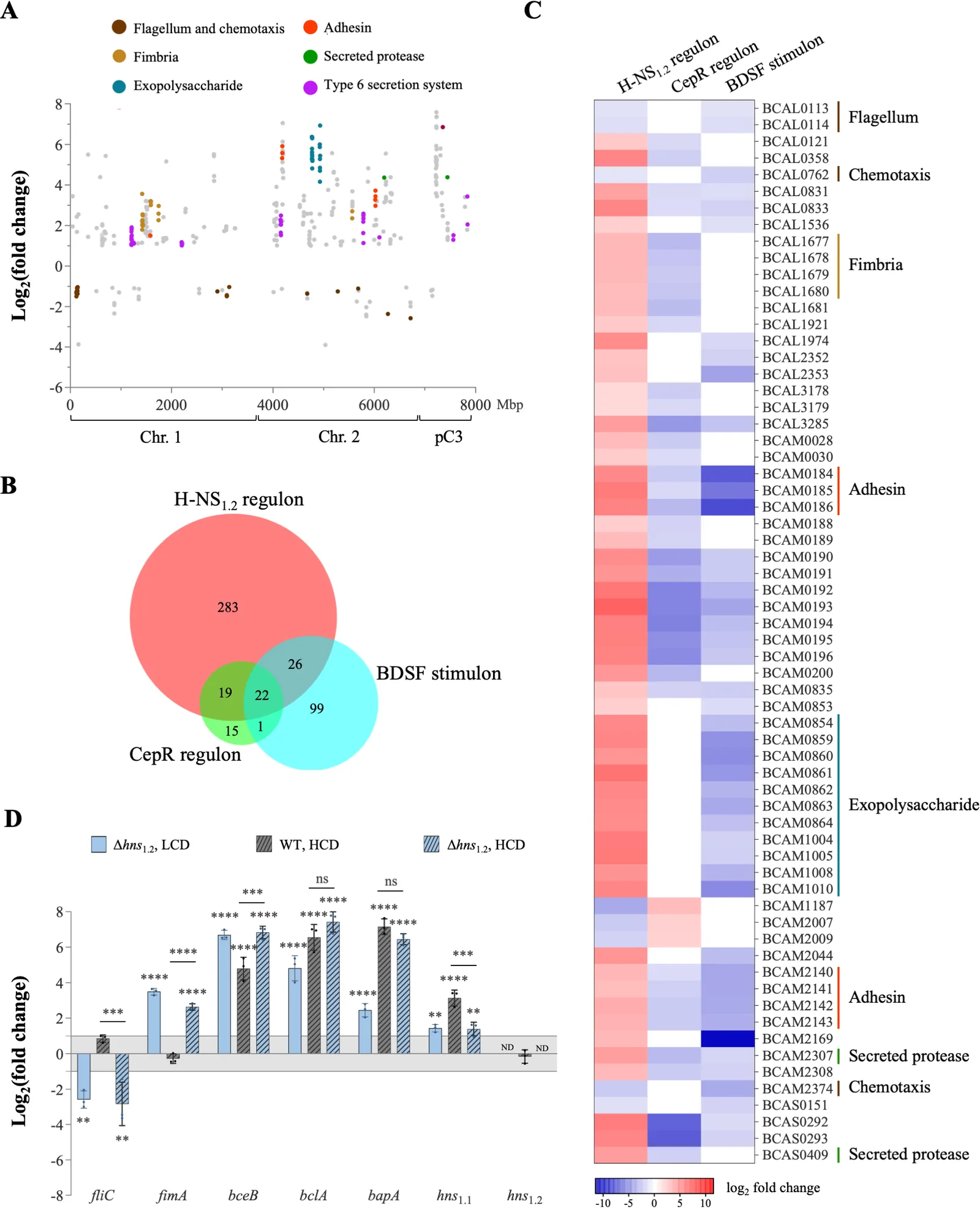
H-NS_1.2_ downregulates critical virulence genes, including several targets of the CepIR and RpfFR quorum-sensing systems. (**A**) Distribution of the H-NS_1.2_-target genes across the three bacterial replicons. All genes considered differentially expressed according to the RNA-seq-based comparative transcriptome analysis (Δ*hns*_1.2_ mutant × parental cells) are shown, highlighting selected virulence genes in different colors. Genes were chosen due to their established or putative involvement in *B. cenocepacia* pathogenicity. Chr. 1, Chr. 2, and pC3 refer to chromosome 1, chromosome 2, and the megaplasmid pC3, respectively, and are included to indicate the replicon in which each gene is located. (**B**) Venn’s diagram comparing the complete set of genes (downregulated and upregulated) whose expression changed upon individually deleting *hns*_1.2_, *cepR*, and *rpfF*. (**C**) Heatmap comparing changes in expression of genes found in at least two out of the three sets of genes shown in panel **B**. Genes are organized according to their position in the bacterial replicons, highlighting several of the same virulence genes depicted in panel **A**. (**D**) Effect of *hns*_1.2_ deletion on transcript levels of selected genes at two different cell densities. Saturated cultures were diluted in fresh LLB medium and grown for 8 h. RT-qPCR experiments were performed starting with total RNA samples obtained from aliquots taken at OD_600nm_ ≃ 0.5 (low cell density [LCD]) and 3.0 (high cell density [HCD]). Results were normalized using the *rpoD* gene as an endogenous control and presented as the log_2_ fold change of the transcript levels relative to parental cells at low cell density. Statistical analysis was carried out using one-way ANOVA followed by Tukey’s test for multiple comparisons. Asterisks indicated above or below a bar indicate significant differences with respect to parental cells at low cell density. For the comparison between the deletion mutant and parental strain at high cell density, either asterisk or ns (no significant change) is indicated above or below a line. (**, *P* < 0.01; ***, *P* < 0.001; ****, *P* < 0.0001). *hns*_1.2_ transcript was not detected in the deletion strain and is denoted as ND.

As the *hns*_1.2_ gene was shown to play a major role in the physiology of *B. cenocepacia* growing in rich medium, we sought to determine the transcriptional changes linked to *hns*_1.2_ deletion. According to an RNA-seq-based comparative transcriptome analysis, 51 and 299 genes were considered downregulated and upregulated, respectively, upon deleting *hns*_1.2_ (Fig. S4A and Table S2). These differentially expressed genes are scattered across the three replicons (Fig. 4A) and account for 5.1% of all predicted genes, suggesting that H-NS_1.2_ globally regulates gene expression in *B. cenocepacia* H111. By analyzing the relative expression of selected genes using real-time quantitative PCR (RT-qPCR), we observed a strong positive correlation between the data obtained from this analysis and the transcriptome data, validating our genome-scale analysis to identify H-NS_1.2_-target genes (Fig. S4B).

Several genes involved in flagellum-dependent motility and chemotaxis were downregulated in the absence of *hns*_1.2_. Particularly, these genes are important for filament assembly, torque generation, and control of flagellar rotation in response to chemicals. All these genes are expected to be expressed in the latter stage of the flagellum assembly (34). In contrast, genes playing a critical role in biofilm formation due to their involvement in adhesion (*bcl* genes coding for lectins and the transcription unit encompassing the adhesin gene *bapA* along with genes involved in BapA secretion through the cell envelope) and in the synthesis of cepacian, one major exopolysaccharide of Bcc (35), showed increased expression in cells lacking *hns*_1.2_ (Fig. 4A; Table S2). Moreover, genes encoding components of three chaperone-usher-type fimbriae and one Flp-type pilus were upregulated in the absence of *hns*_1.2_ (Fig. 4A; Table S2). Because operons and clusters involved in the synthesis of other known or predicted fimbriae and exopolysaccharides had their expression levels unchanged in our transcriptome analysis, H-NS_1.2_ targets specific adhesion and exopolysaccharide genes in *B. cenocepacia* H111.

The *zmpA* and *zmpB*, which encode important secreted proteases (36, 37), were included in the list of H-NS_1.2_-controlled genes (Fig. 4A; Table S2). Our transcriptome data also showed upregulation of a specific part of a gene cluster coding for a type VI secretion system (T6SS), mainly genes involved in forming the contractile sheath, the Hcp tube, and a VgrG protein (Fig. 4A; Table S2). Additionally, *vgrG* and associated potential effector and immunity genes distributed along the two chromosomes are upregulated in the Δ*hns*_1.2_ strain (Fig. 4A; Table S2). On the other hand, none of the genes on chromosome 1 that are related to a previously characterized antibacterial T6SS (38) showed altered expression in the Δ*hns*_1.2_ strain. Therefore, our data imply that H-NS_1.2_ downregulates genes encoding proteins that are possibly secreted by T6SS. Moreover, genes involved in killing *C. elegans* (*aid* genes [39]) and fungi (*afc* genes [40]) were also assigned as H-NS_1.2_ targets (Table S2).

A noteworthy observation was the *hns*_1.1_ upregulation in the absence of *hns*_1.2_, suggesting a regulatory cascade involving these *hns* genes (Table S2). Furthermore, other genes involved in gene expression regulation were found upregulated in the Δ*hns*_1.2_ strain relative to parental cells (Table S2), implying an even more complex regulatory network controlled by H-NS_1.2_. Interestingly, although neither *cepIR* nor *rpfFR* was affected by *hns*_1.2_ deletion, we found several H-NS_1.2_-target genes among those controlled by one or two of the quorum-sensing (QS) systems of *B. cenocepacia* H111 (Fig. 4B). A total of 57 genes were previously identified as comprising the CepR regulon (8), which is activated in response to increased concentration of CepI-produced *N*-acyl homoserine lactone molecules, and our analysis showed that 72% of the CepR-dependent genes are included in the list of genes regulated by H-NS_1.2_ (Fig. 4B). Notably, all co-regulated genes are affected by H-NS_1.2_ and CepR in an opposite way (Fig. 4C). Our analysis also identified a substantial overlap between the H-NS_1.2_ regulon and the *Burkholderia* diffusible signal factor (BDSF, *cis*-2-dodecenoic acid) stimulon (7) (Fig. 4B). Additionally, almost all the co-regulated genes are impacted by H-NS_1.2_ and BDSF in a divergent manner (Fig. 4C). Therefore, this comparative analysis shows that several virulence genes are controlled by H-NS_1.2_ and at least one of the QS systems in *B. cenocepacia* H111.

As H-NS_1.2_, but not the QS systems, impacts the co-regulated genes at low cell density, we investigated the extent to which H-NS_1.2_ contributes to the expression of these genes at high cell density, when the QS systems are active in *B. cenocepacia*. Using RT-qPCR, we observed that *fliC* and *fimA* were not differentially expressed in wild-type cells upon comparing the transcript levels at high cell density (OD_600nm_ around 3.0) to those at low cell density (OD_600nm_ around 0.5). Moreover, *fliC* and *fimA* were affected by *hns*_1.2_ deletion to the same extent at both cell densities (Fig. 4D). Similarly, *hns*_1.2_ transcript levels in wild-type cells were quite the same at low and high cell densities (Fig. 4D). On the other hand, *bceB*, *bclA*, *bapA*, and *hns*_1.1_ were all upregulated in wild-type cells upon increasing cell density, but only a small or no further increase in their expression was observed at high cell density when *hns*_1.2_ was absent (Fig. 4D). Together, these data suggest a remarkable decrease in the contribution of *hns*_1.2_ to control the expression of genes co-regulated with the QS systems at high cell density in *B. cenocepacia*.

### *hns*_3.1_ affects gene expression in a way opposite to *hns*_1.2_

**Fig 5:**
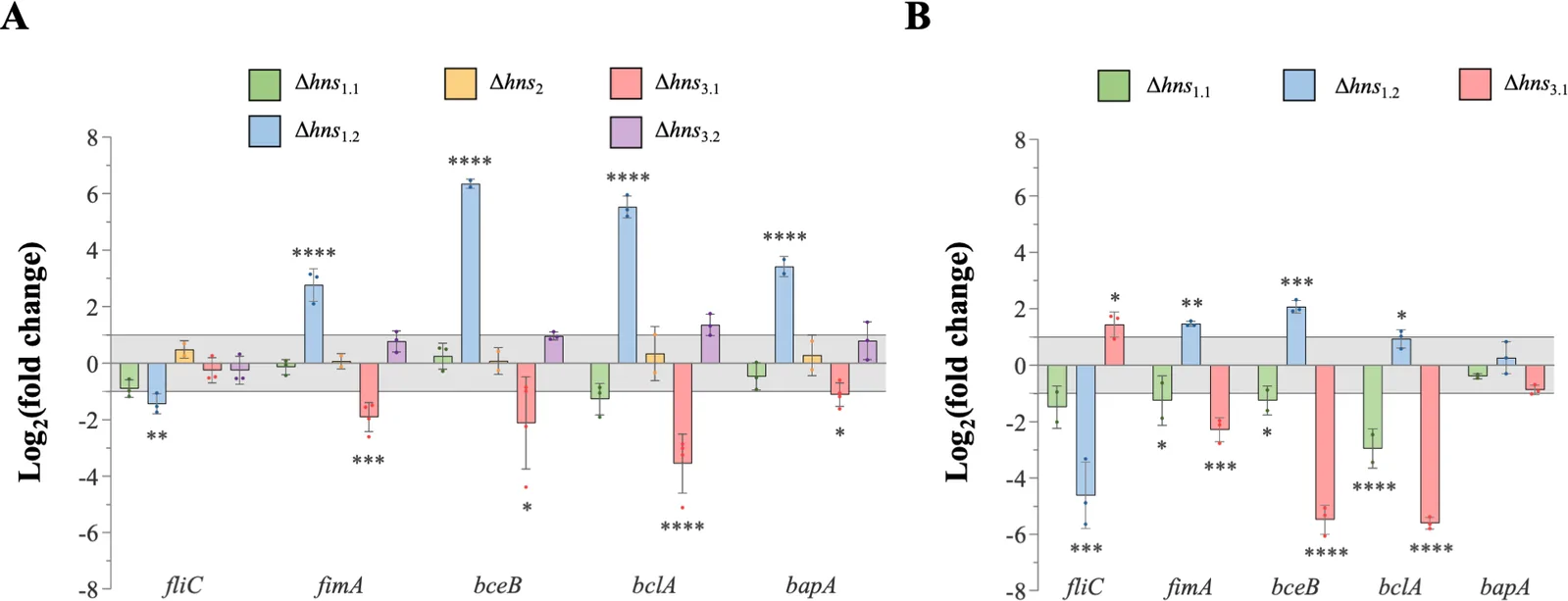
Effect of the *hns* genes on the transcript levels of selected targets in two different growth media. Saturated cultures were diluted in either LLB (**A**) or M9 (**B**) and grown to exponential phase (OD_600nm_ ≃ 0.5). RT-qPCR experiments were performed starting with total RNA samples, and results were normalized using the *rpoD* gene as an endogenous control and presented as the log_2_ fold change of the transcript levels in an *hns* deletion mutant relative to parental cells. Data are mean values ± standard deviations of two to four independent experiments. Statistical analysis was carried out using one-way ANOVA followed by Dunnett’s test to compare the mutant strains to a single control (WT) (*, *P* < 0.05; **, *P* < 0.01; ***, *P* < 0.001; ****, *P* < 0.0001).

To investigate the relative impact of the other H-NS proteins on gene expression, we compared transcript levels of selected genes in each mutant strain with those of parental cells by RT-qPCR. Cells deleted for *hns*_3.1_ displayed decreased expression of *fimA*, *bceB*, *bclA*, and *bapA*, contrasting with the upregulation observed for all these genes in the Δ*hns*_1.2_ strain (Fig. 5A). On the other hand, the absence of *hns*_1.1_ and *hns*_3.2_ led to only a minor impact on gene expression (Fig. 5A), which could be related to the presence of *hns*_1.2_ masking the effect of other *hns* genes. Our analysis showed no other case in which *hns* genes regulate one another apart from the *hns*_1.2_-mediated downregulation of *hns*_1.1_ (Fig. S5). Therefore, by analyzing the expression of selected genes, the results support an assumption that *hns*_1.2_ is the major *hns* gene in cells growing in a rich medium and shed light on an opposite role played by *hns*_3.1_.

Because our phenotypic analyses revealed a growth medium-dependent impact of *hns*_1.2_ and *hns*_3.1_ on biofilm formation, we analyzed the relative expression of the same genes in cells grown in minimal medium. Compared with results obtained in rich medium, the absence of *hns*_1.2_ caused a lesser impact on the expression of *fimA*, *bceB*, *bclA*, and *bapA* (Fig. 5B). On the other hand, *hns*_3.1_ deletion led to an even more drastic reduction in the transcript levels of *fimA*, *bceB*, and *bclA* (Fig. 5B). Moreover, *fliC* was upregulated in cells deleted for *hns*_3.1_ (Fig. 5B), further supporting the greater impact of this *hns* gene on gene expression regulation in cells grown in minimal medium. Together, our results show that the extent to which *hns*_1.2_ and *hns*_3.1_ affect gene expression changes according to the growth medium, in agreement with the relative impact of these *hns* genes on biofilm formation (Fig. 2C). We also compared Δ*hns*_1.1_ and parental cells in minimal medium and found decreased expression of *fimA*, *bceB*, and *bclA* in the mutant strain (Fig. 5B). Thus, our data also suggest an increased contribution of *hns*_1.1_ to gene expression in minimal medium.

### H-NS proteins produced by means of a replicative plasmid affect gene expression, motility, and biofilm formation

**Fig 6:**
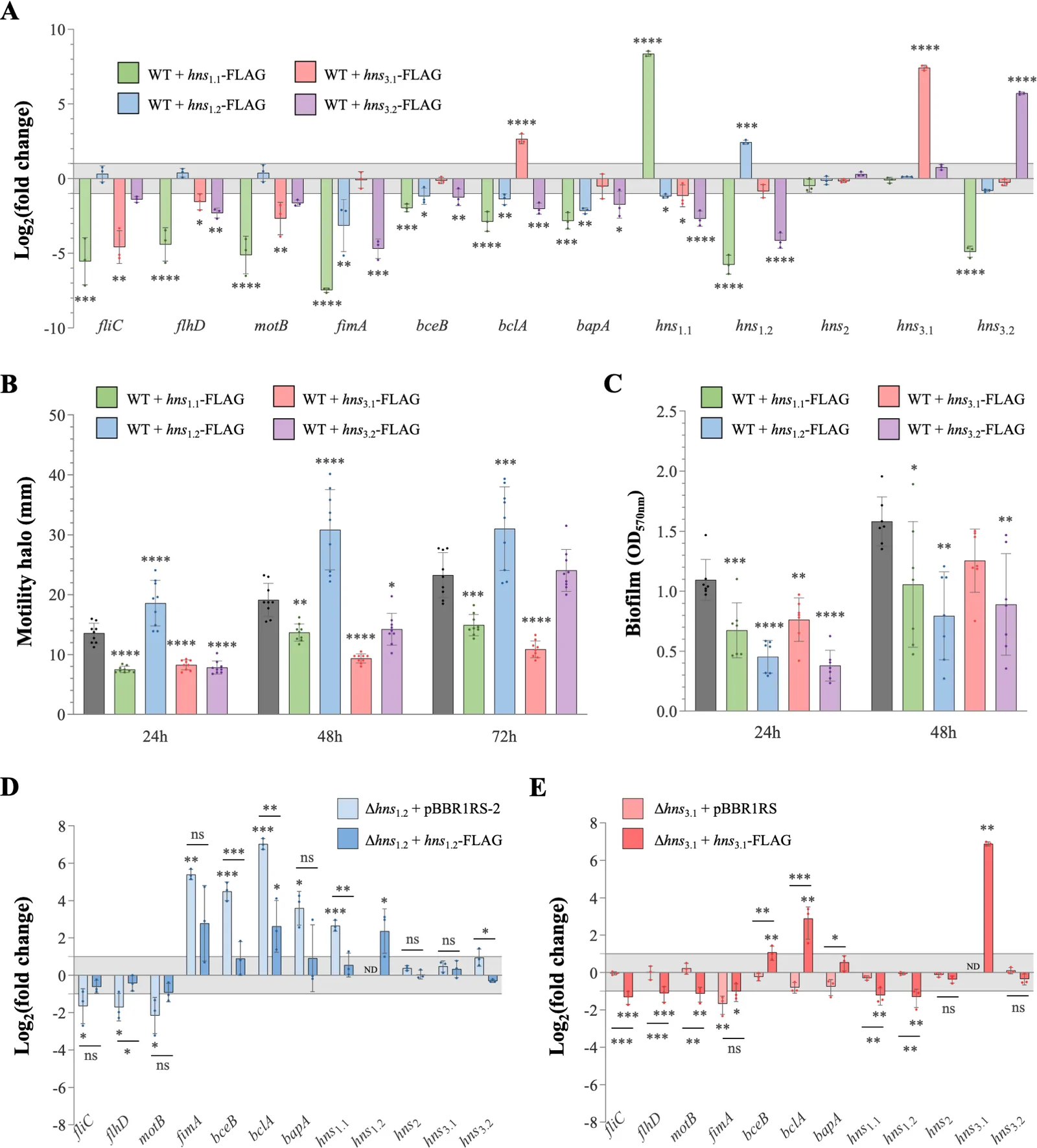
Characterization of *B. cenocepacia* cells carrying a plasmid-encoded *hns* gene reinforces the opposite roles of *hns*_1.2_ and *hns*_3.1_ in gene expression and shows that *hns*_1.1_ and *hns*_3.2_ control several H-NS_1.2_-target genes. (**A**) Effect of a plasmid-encoded *hns* gene on transcript levels of selected genes in the wild-type cells. Saturated cultures were diluted in fresh LLB medium supplemented with 0.5% rhamnose and grown to exponential phase (OD_600nm_ ≃ 0.5). RT-qPCR experiments were performed starting from total RNA samples, and results were normalized using the *rpoD* gene as an endogenous control and presented as the log_2_ fold change of the transcript levels in an *hns* overexpression strain relative to parental cells harboring the empty vector. Data are mean values ± standard deviations of three independent experiments. In addition to the genes analyzed in the deletion mutants, we also included *motB* (downregulated in the Δ*hns*_1.2_ strain) and *flhD* (not considered differentially expressed between the *hns*_1.2_ mutant and parental cells but located upstream of *motAB*) in the analysis to offer a broader view of the effect of H-NS overproduction on flagellum-related genes. Statistical analysis was carried out using one-way ANOVA followed by Dunnett’s test to compare the mutant strains to a single control (WT + pBBR1 RS) (*, *P* < 0.05; **, *P* < 0.01; ***, *P* < 0.001; ****, *P* < 0.0001). (**B**) Swarming motility of wild-type cells harboring an extra, plasmidial copy of an *hns* gene or the empty vector. Saturated cultures were diluted in fresh LLB medium supplemented with 0.5% rhamnose, grown to OD_600nm_ = 0.4, and spotted on rhamnose-containing semisolid plates. The diameter of the motility halo was determined at the indicated time points, and data are mean values ± standard deviations of three independent experiments with three technical replicates each. (**C**) Biofilm formation of wild-type cells harboring an extra, plasmidial copy of an *hns* gene or the empty vector. Saturated cultures were diluted in fresh LLB medium supplemented with 0.5% rhamnose and grown in microtiter polystyrene dishes under static conditions. At the indicated time points, attached cells were stained with 1% crystal violet, and OD_570nm_ was determined. Data are mean values ± standard deviation of three independent experiments, with two to three technical replicates each. Notably, biofilm measured at 48 h showed considerable variation, which may be related to disparity in H-NS levels after prolonged time in the presence of rhamnose. (D and E) Effect of a plasmid-encoded *hns* gene on transcript levels of selected genes in *hns* deletion cells. Saturated cultures were diluted in fresh LLB medium supplemented with 0.5% rhamnose and grown to exponential phase (OD_600nm_ ≃ 0.5). RT-qPCR experiments were performed starting from total RNA samples, and results were normalized using the *rpoD* gene as an endogenous control and presented as the log_2_ fold change of the transcript levels relative to parental cells harboring an empty vector (pBBR1RS in panel D and pBBR1RS-2 in panel** E**). Data are mean values ± standard deviations of three independent experiments. Statistical analysis was carried out using one-way ANOVA followed by Tukey’s test for multiple comparisons. An asterisk indicated above or below a bar indicates a significant difference with respect to parental cells harboring an empty vector. For the comparison of the deletion mutant carrying the plasmid-encoded *hns* gene with the same mutant strain harboring the empty vector, either an asterisk or ns (no significant change) is indicated above or below a line. (*, *P* < 0.05; **, *P* < 0.01; ***, *P* < 0.001). Two-tailed, unpaired Student’s test was used while analyzing the transcript levels of the *hns* gene overexpressed in the cell. Transcript of an *hns* gene was not detected in the corresponding deletion mutant carrying an empty vector and is denoted as ND.

To further elucidate the contribution of the H-NS proteins to gene expression in *B. cenocepacia*, we created cells to individually produce the H-NS proteins fused to the FLAG tag at their C-termini by means of a replicative plasmid that we constructed in this study for rhamnose-inducible expression (see the Materials and Methods section for details). Upon induction, we found that the strain created to produce H-NS_2_-FLAG could not further increase its biomass (Fig. S6A) and underwent a decrease in viability, which was sustained for at least 10 h (Fig. S6B). This observation suggests that increased expression of *hns*_2_ may be toxic for *B. cenocepacia*, preventing us from using this strain for the expression and phenotypic analyses. Conversely, the bacterium was able to tolerate expression of the other *hns* genes cloned in the same replicative plasmid, showing a growth delay only when H-NS_3.2_-FLAG was produced (Fig. S6A). For all these strains, the transcript levels of a specific *hns* gene increased under the inducing condition in cells harboring the plasmidial copy of the same gene (Fig. 6A), suggesting that this set of strains most probably overproduce H-NS proteins. H-NS_1.2_-FLAG and H-NS_3.1_-FLAG were produced at quite similar levels under inducing conditions (Fig. S6C) but led to different effects on the expression of genes involved in motility and biofilm formation. Notably, H-NS_3.1_-FLAG downregulated genes that were not affected by H-NS_1.2_-FLAG (*fliC*, *flhD*, *motB*, all genes involved in motility) but upregulated or did not impact genes that were downregulated by H-NS_1.2_-FLAG (*fimA*, *bceB*, *bclA*, and *bapA*, three of them with established roles in biofilm formation [8, 41]) (Fig. 6A). These observations reinforce that H-NS_1.2_ and H-NS_3.1_ regulate gene expression in an opposite way in *B. cenocepacia* H111. On the other hand, the plasmidial copies of *hns*_1.1_ and *hns*_3.2_ led to higher levels of the corresponding FLAG-tagged proteins under the inducing condition (Fig. S6C) and impacted the expression of H-NS_1.2_-dependent genes to greater extents, downregulating genes associated with both motility and biofilm formation (Fig. 6A).

By investigating the effect of producing a specific FLAG-tagged protein on the expression of the other *hns* genes, we observed that H-NS_1.1_-FLAG downregulated *hns*_3.2_; H-NS_3.2_-FLAG downregulated *hns*_1.1_; and both proteins led to a reduction in the *hns*_1.2_ transcript levels (Fig. 6A). Additionally, H-NS_1.2_-FLAG and H-NS_3.1_-FLAG led to decreased *hns*_1.1_ transcript levels (Fig. 6A). In contrast, the transcript levels of both *hns*_2_ and *hns*_3.1_ were only affected when the plasmidial copy of the same gene was introduced into the cells (Fig. 6A). Thus, our data support that *hns*_1.1_, *hns*_1.2_, and *hns*_3.2_ are all targets for H-NS-mediated control in *B. cenocepacia* H111.

Consistent with the decreased expression of flagellar genes, reduced swarming motility was observed in strains producing either H-NS_1.1_-FLAG or H-NS_3.1_-FLAG when compared with parental cells carrying the empty vector (Fig. 6B; Fig. S6D). Curiously, when grown under static conditions, these strains displayed aggregate formation at the bottom of the wells (Fig. S6E). Conversely, H-NS_1.2_-FLAG production led to increased motility (Fig. 6B; Fig. S6D) and decreased aggregate formation (Fig. S6E). We also observed decreased amounts of biofilm upon producing one of the following proteins in the bacterium cells: H-NS_1.1_-FLAG, H-NS_1.2_-FLAG, and H-NS_3.1_-FLAG (Fig. 6C). We performed the same phenotypic analyses with the H-NS_3.1_-FLAG-producing strain. However, despite decreased motility and biofilm formation, the results are difficult to interpret because at least part of the effects may be linked to the growth defect (Fig. 6B and C). Together, these phenotypic analyses using strains carrying an extra, plasmidial copy of *hns* corroborate the involvement of *hns*_1.1_, *hns*_1.2_, and *hns*_3.1_ in the control of motility and biofilm formation in *B. cenocepacia* H111.

We also asked whether the presence of a plasmid-encoded *hns* gene could rescue the effect of deleting the corresponding chromosomal *hns* gene. Surprisingly, we found that the Δ*hns*_1.2_ strain grows in the presence of chloramphenicol, preventing us from using the same plasmid employed to investigate the effect of overexpressing *hns*_1.2_ in wild-type cells. To circumvent this challenge, we created a similar replicative plasmid and a derivative construct harboring *hns*_1.2_, both with a kanamycin resistance marker. Upon expressing *hns*_1.2_ by means of this replicative plasmid in the *hns*_1.2_ mutant, we observed that the transcripts of *fimA*, *hns*_1.1_, and the genes involved in motility and biofilm formation were restored to near-normal levels as found in the wild-type cells carrying the empty vector (Fig. 6D). Accordingly, the plasmid-encoded *hns*_1.2_ gene reverted all the following phenotypes linked to *hns*_1.2_ deletion: (i) slow growth, as revealed by the colony size; (ii) whitish appearance; (iii) cellular aggregation under static conditions; (iv) biofilm adhered to an abiotic surface; and (v) swarming motility (Fig. S7A through C).

Curiously, only *fimA* was found to be downregulated in the *hns*_3.1_ mutant relative to parental cells when both strains were grown in complex medium supplemented with rhamnose (Fig. 6E), suggesting that *hns*_3.1_ has almost no impact on the expression of the genes analyzed under this growth condition. However, the presence of a plasmid-encoded *hns*_3.1_ gene in the mutant strain resulted in downregulation of *hns*_1.1_, *hns*_1.2_, and the motility genes and upregulation of *bceB* and *bclA* with respect to both the wild-type and mutant cells carrying the empty plasmid (Fig. 6E). Consistent with the observation that the plasmid-encoded *hns*_3.1_ led to transcriptional changes in the Δ*hns*_3.1_ strain similar to those of wild-type cells carrying the same plasmid, we observed that the Δ*hns*_3.1_ mutant harboring the plasmid-encoded *hns*_3.1_ but not the mutant with the empty vector was defective in motility (Fig. S7D). Likewise, the Δ*hns*_1.1_ mutant harboring a plasmid-encoded *hns*_1.1_ gene behaved similarly to wild-type cells carrying the same plasmid with respect to swarming motility, but no phenotypic change was observed for the *hns*_1.1_ deletion mutant growing in the presence of rhamnose (Fig. S7D). Therefore, despite not being possible to determine the ability of the plasmidial copy of *hns*_1.1_ and *hns*_3.1_ to revert motility in the corresponding mutants under the conditions analyzed, the opposite phenotypes observed as a function of the gene copy number (compared Fig. 2 and 6) suggest that the phenotypic changes observed in the *hns*_1.1_ and *hns*_3.1_ deletion mutants most probably are linked to the specific genetic manipulations.

## DISCUSSION

In this work, we analyzed the relative contribution of the *hns* genes to *B. cenocepacia* H111 physiology and pathogenicity. Our phenotypic and transcriptome analyses support a clear motile-to-biofilm lifestyle switch in cells lacking *hns*_1.2_. Among the genes upregulated in these cells, *bapA* and the *bclACB* operon, which code for surface proteins, and the cepacian biosynthesis genes *bceD* and *bceF* were demonstrated to contribute to biofilm formation (8, 41). Moreover, genes on the 24-kbp region on the megaplasmid were found to play important roles in biofilm formation in *B. cenocepacia* (42). Therefore, the increased ability of the Δ*hns*_1.2_ strain to form biofilm is most likely the result of the combined action of several upregulated genes. As a member of the H-NS-family protein, H-NS_1.2_ is expected to directly bind to and control genes involved in biofilm formation. Moreover, the presence of genes coding for transcriptional regulators among the H-NS_1.2_-dependent genes would also imply that a subset of the genes impacted by *hns*_1.2_ deletion is probably under the direct control of these regulatory genes. Remarkably, the 24-kbp region on the megaplasmid is positively regulated by its adjacent gene, *shvR* (42), and both *gtrR* and a newly discovered gene coding for a LysR transcriptional factor control the expression of many genes involved in biofilm formation in *B. cenocepacia* (9, 10). Similarly, increased levels of the LysR-family regulator LdhR in *B. cenocepacia* cells lacking *hns*_1.2_ could directly impact the expression of other genes, leading these cells to aggregate, as *ldhR* was shown to play a role in planktonic cellular aggregation in *B. multivorans* (12).

The biofilm lifestyle is crucial for the bacterial persistence in the host and establishment of a chronic infection. Recently, *hns*_1.2_ was found as a detrimental gene for *B. cenocepacia* H111 while colonizing the pig bronchiole tissue, which is considered an *ex vivo* model for chronic infection (43). Due to the increased expression of the genes mentioned above, cells disrupted in their ability to produce H-NS_1.2_ may attach to and develop into a biofilm structure on bronchiole tissue more efficiently than wild-type cells. The increased levels of the FimA fimbrial protein could also contribute to the enhanced colonization of bronchiole tissue by Δ*hns*_1.2_ cells, as insertional mutations in both *fimA* and the chaperone gene involved in FimA export through the cell envelope decreased the bacterial fitness to colonize the tissue (43). Therefore, despite proving to be dispensable for biofilm formation on abiotic surfaces (8), *fimA* appears to play an important role in attachment or biofilm formation in host tissues. Different from this clear improved fitness of Δ*hns*_1.2_ cells in colonizing an animal model for chronic infection, no significant change in the virulence of *B. cenocepacia* H111 was associated with *hns*_1.2_ disruption in our experiments using *G. mellonella*, an animal model for acute infection. This observation implies that the physiological changes in the mutant strain neither confer additional protection nor render the bacterium more vulnerable to the immune system.

The motile-to-biofilm lifestyle transition can be driven by an increase in the c-di-GMP concentration. However, this molecule does not seem to reach high levels in Δ*hns*_1.2_ cells, as genes regulated by the c-di-GMP-responsive protein BerA (44) are not affected in this mutant. On the other hand, most of the genes controlled by the CepIR QS system (8) are included in the list of genes regulated by H-NS_1.2_, suggesting that CepR may act as a counter-silencing factor. By binding with higher affinity to several H-NS_1.2_-target sites, CepR would dissociate H-NS_1.2_ from DNA, a mechanism similar to that proposed for *Vibrio cholerae* LuxR in response to increased cell density (23). In addition, our data could also imply the involvement of a second QS system, which is triggered by the BDSF molecule, in releasing genes from the H-NS_1.2_-mediated silencing. This assumption relies on the fact that the BDSF-sensing protein, RpfR, in addition to co-regulating several genes with CepR, also controls specific targets (7), such as the *bclACB* operon and the two gene clusters for cepacian biosynthesis observed among the H-NS_1.2_-dependent genes. Our gene expression analysis showing that *hns*_1.2_ is not affected by an increase in cell density but shows a reduced impact on the expression of QS-regulated genes under this condition also agrees with a possible functional interplay between H-NS_1.2_ and the QS systems in *B. cenocepacia*. Though QS activation may explain the change in the expression of a subset of the H-NS_1.2_-target genes when the bacterium switches to the biofilm-associated lifestyle, the altered expression of several other genes, such as T6SS genes, is left without any hypothesis, suggesting that other counter-silencers or even additional mechanisms exist to dissociate H-NS_1.2_ from DNA in *B. cenocepacia* H111.

All phenotypic and gene expression analyses shown in this study were carried out in standard laboratory media and normoxia conditions, which considerably deviate from the natural conditions the bacterium encounters in the airways of cystic fibrosis patients. A previously reported transcriptome analysis with *B. cenocepacia* cells showed that supplementation of a minimal medium with 10% cystic fibrosis sputum caused upregulation of genes putatively involved in adhesion, including *bapA* (45). In another study, expression of the *bcl* genes was upregulated during the long-term adaptation of *B. cenocepacia* to cystic fibrosis patients’ airways (46). Furthermore, genes involved in cepacian biosynthesis were upregulated, and the amount of adhered biomass and pellicle formation were increased in *B. cenocepacia* H111 growing under micro-oxia conditions (47). Notably, the fold change in the transcript levels of *hns*_1.2_ was not twofold or higher under any of the abovementioned conditions, suggesting that the upregulation of biofilm-related genes may not be linked to a decreased expression of *hns*_1.2_. Instead, increased expression of such genes could be explained at least in part by H-NS_1.2_ dissociation from these genes due to a conformational change or by the action of counter-silencing factors in response to specific conditions found in the cystic fibrosis patients’ airways.

Like the *hns*_1.2_ gene, *hns*_1.1_ was also found to be important for motility and detrimental for biofilm in our phenotypic analyses. However, while the reduced motility was observed under the same condition for both mutant strains, increased biofilm formation was noticed under conditions that were specific for each mutant. In cells growing in a complex medium, the absence of *hns*_1.1_ did not affect the cell’s ability to attach to abiotic surfaces. Considering that an extra, plasmidial copy of *hns*_1.1_ leads to downregulation of genes involved in biofilm formation and impairs the cell’s ability to form biofilm in a complex medium, we predict that *hns*_1.1_ is not expressed in wild-type cells at levels high enough to cause changes in the biofilm when deleted. On the other hand, the loss of *hns*_1.1_ led to increased biofilm formation in a minimal medium, a condition in which *hns*_1.2_ deletion had no effect on biofilm. Therefore, a possible change in the relative levels and/or post-translational modification status of H-NS_1.1_ and H-NS_1.2_ could explain the change with respect to the *hns* gene playing the major role in the control of biofilm formation in a minimal medium compared to a complex medium. The simplest explanation would consider H-NS_1.2_ dissociation from DNA, leading to *hns*_1.1_ upregulation. Notably, the relative impact of *hns*_1.1_ and *hns*_1.2_ on the biofilm formation in a minimal medium is in accordance with the fact that *hns*_1.1_ but not *hns*_1.2_ plays a role in bacterial virulence in the *G. mellonella* infection model. Additionally, the increased and decreased contributions of *hns*_1.1_ and *hns*_1.2_, respectively, to gene expression regulation in minimal medium also support the results in *G. mellonella*.

As judged by the observation that *hns*_3.2_ affects neither motility nor biofilm under any of the conditions tested, this gene seems to be expressed at levels even lower than those of *hns*_1.1_ in wild-type cells. Nonetheless, a clear capability of H-NS_3.2_ to downregulate H-NS_1.2_-target genes was observed using an overproducing strain, suggesting that *hns*_3.2_ could play a considerable role in global gene expression under conditions in which its expression is increased. Similarly, *hns*_2_ deletion caused no effect on motility and biofilm. Even though the possibility that high H-NS_2_ levels could silence gene expression was not accessed due to the toxic effect arising from overproducing this protein, our *in silico* analysis provides evidence that H-NS_2_ binds DNA by means of a conserved AT-hook motif. A closer inspection of available transcriptome and proteome data showed upregulation of *hns*_3.2_ under oxidative stress induced by inorganic peroxide and during the stationary growth phase in a minimal medium (48). In addition, H-NS_2_ levels increased when cells were grown under nitrogen-limited conditions (49). Therefore, under these conditions, *hns*_2_ and *hns*_3.2_ may show a certain impact on the motile-to-biofilm lifestyle switch.

Interestingly, we found that *hns*_3.1_ positively regulates genes involved in biofilm formation and showed that cells deleted for this gene display increased motility and decreased amount of biofilm. Therefore, these results contrast with the upregulation of the same genes, the reduced motility, and the increased biofilm formation observed in cells lacking *hns*_1.2_. As the transcript levels of *hns*_1.2_ are not affected by *hns*_3.1_ deletion, one possible explanation is that H-NS_3.1_ negatively regulates the ability of H-NS_1.2_ to silence gene expression. Our *in silico* analysis revealed that both H-NS_1.2_ and H-NS_3.1_ may have functional dimerization sites 1 and 2, which would allow not only self-association in homooligomers but also interaction of one protein with another into heterooligomers. In contrast, a proline residue in a position predicted to directly contact DNA may reduce the affinity of H-NS_3.1_-containing heterooligomers to DNA. In this scenario, the action of counter-silencers is facilitated. Moreover, an increase in the H-NS_3.1_ levels, leading to a further decrease in the heterooligomer affinity to DNA, corresponds to another mechanism to release genes from H-NS_1.2_-mediated silencing. The above proposed assumption that H-NS_1.2_ dissociates from DNA in cells growing in a minimal medium could explain both the decreased biofilm formation and the attenuated virulence of cells lacking *hns*_3.1_. In the absence of *hns*_3.1_, H-NS_1.2_ dissociation from DNA and the motile-to-biofilm switch are both compromised. Therefore, H-NS_3.1_ would serve to modulate H-NS_1.2_-mediated gene expression. Regulation of H-NS proteins by a truncated H-NS version capable of heteroassociation but impaired in DNA binding due to the absence of a DNA-binding domain has been reported (50). Nonetheless, here we present a full-length H-NS protein whose DNA-binding activity seems to be affected by an amino acid substitution in a critical position for binding DNA.

**Fig 7:**
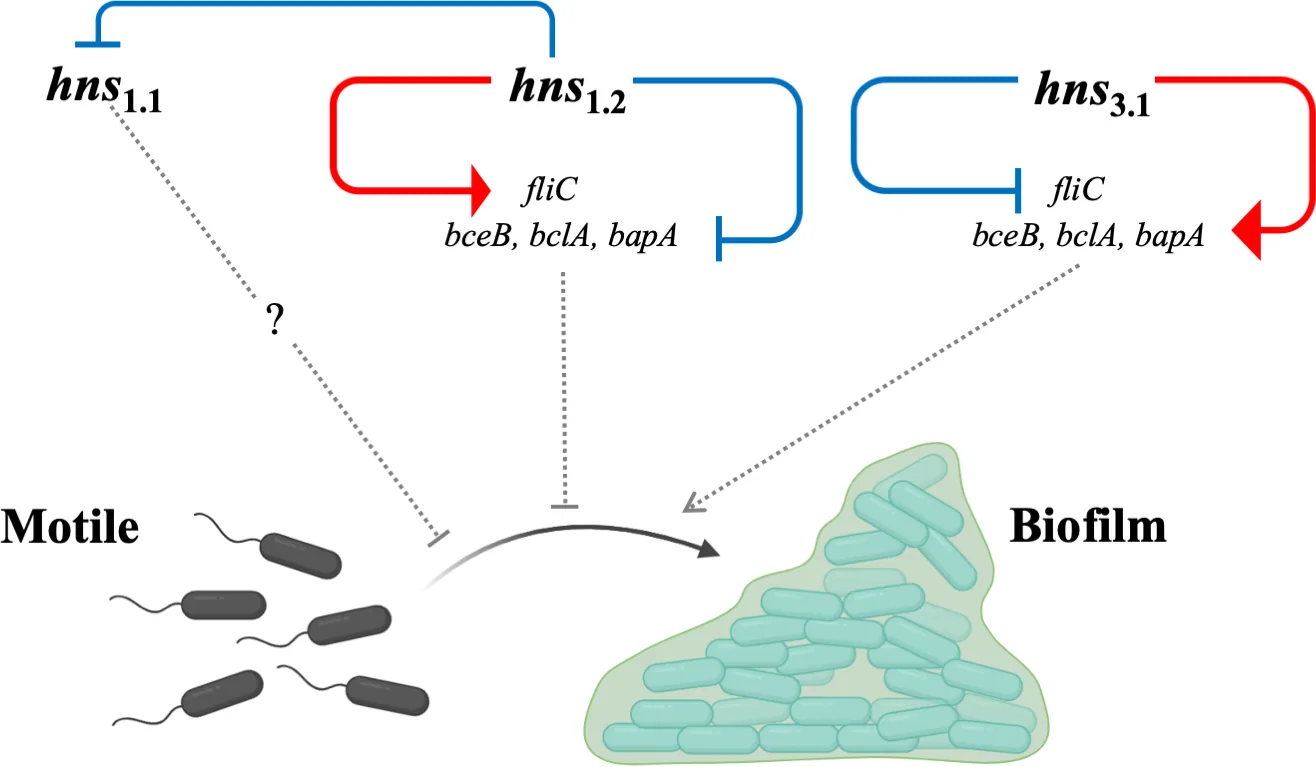
Model for the control of the motile-to-biofilm switch in *B. cenocepacia* H111. Schematic representation of the transition from the motile and biofilm-associated lifestyle, underlining the role of different *hns* genes and their impact on the expression of selected genes.

While increased expression of *hns*_1.2_ and *hns*_3.1_ resulted in downregulation of genes involved in biofilm formation and motility, respectively, overexpression of *hns*_1.1_ and *hns*_3.2_ negatively impacted the expression of both gene groups. This observation may imply some diversification in the ability of the H-NS proteins to regulate the expression of genes involved in biofilm formation and motility in *B. cenocepacia* H111, which was also evident upon analyzing the deletion mutants. Our data suggest a model in which *hns*_1.1_ and *hns*_1.2_ work to maintain cells at the motile stage, while *hns*_3.1_ promotes a lifestyle transition to biofilm (Fig. 7). While the impact of *hns*_1.2_ and *hns*_3.1_ on the expression of genes involved in biofilm formation and motility is consistent with their role in the motile-to-biofilm lifestyle transition, the increased biofilm formation in the *hns*_1.1_ mutant cannot be explained, considering the effect of *hns*_1.1_ on the expression of specific genes, such as *bceB*, *bclA*, and *bapA*, suggesting that other H-NS_1.1_-dependent genes may be responsible for the increased biofilm formation in the mutant strain.

Considering the previous studies that characterized *hns* mutants in Bcc species, we conclude that genes controlled by H-NS proteins are conserved in at least two members of this bacterial group, namely, *B. cenocepacia* and *B. multivorans*. In addition, a regulatory cascade of *hns* genes exists in these two species. Curiously, *B. multivorans* carries three putative *hns* genes coding for proteins with a proline residue in the expected critical position for DNA binding, and none of them are part of the *hns* regulatory cascade (16). Therefore, this observation implies that H-NS_3.1_ in *B. cenocepacia* H111 is not the result of point mutation in an *hns* gene in this bacterium. Rather, H-NS_3.1_ seems to represent a conserved molecular mechanism to control the association of other H-NS proteins with the nucleoid and modulate gene expression apart from the *hns* regulatory cascade. By systematically investigating the contribution of all five *hns* genes to *B. cenocepacia* H111 physiology and pathogenicity, we considerably expand our understanding about the functional interplay of H-NS proteins in the *Burkholderia cepacia* complex.

## MATERIALS AND METHODS

### Bacterial strains and growth conditions

Strains used in this study are described in Table 1. *B. cenocepacia* strains were grown in Lenox LB medium (LLB, 1% tryptone, 0.5% yeast extract, and 0.5% NaCl) or M9 medium (0.5% glucose, 0.05% NaCl, 0.6% Na_2_HPO_4_, 0.3% KH_2_PO_4_, 0.1% NH_4_Cl, 1 mM MgSO_4_, and 0.1 mM CaCl_2_) at 22°C–25°C or 37°C. *E. coli* strains were grown in LB medium (1% tryptone, 0.5% yeast extract, and 1% NaCl) at 37°C. When appropriate, the medium was supplemented with 0.5% glucose, 0.5% rhamnose, kanamycin (50 μg/mL), trimethoprim (50 μg/mL), gentamicin (20 μg/mL), chloramphenicol (30 μg/mL), irgasan (25 μg/mL), and 1.5% agar (for solid media).

**TABLE 1:** Strains and plasmids

Strain/plasmid	Description	Reference
Strain		
*Burkholderia cenocepacia*		
WT	CF isolate, Germany	(51)
WT + *hupA*::pGPI*hupA*-FLAG	H111 derivative harboring the pGPI-SceI_*hupA*-FLAG plasmid	This study
WT + *hns*_1.1_::pGPI*hns*_1.1_-FLAG	H111 derivative harboring the pGPI-SceI_*hns*_1.1_-FLAG plasmid	This study
Δ*hns*_1.1_	*hns*_1.1_ in-frame deletion mutant	This study
Δ*hns*_1.2_	*hns*_1.2_ in-frame deletion mutant	This study
Δ*hns*_2_	*hns*_2_ in-frame deletion mutant	This study
Δ*hns*_3.1_	*hns*_3.1_ in-frame deletion mutant	This study
Δ*hns*_3.2_	*hns*_3.2_ in-frame deletion mutant	This study
WT + pBBR1RS	H111 derivative harboring the pBBR1RS plasmid	This study
WT + *hns*_1.1_-FLAG	H111 derivative harboring the pBBR1RS_*hns*_1.1_-FLAG plasmid	This study
WT + *hns*_1.2_-FLAG	H111 derivative harboring the pBBR1RS_*hns*_1.2_-FLAG plasmid	This study
WT + *hns*_2_-FLAG	H111 derivative harboring the pBBR1RS_*hns*_2_-FLAG plasmid	This study
WT + *hns*_3.1_-FLAG	H111 derivative harboring the pBBR1RS_*hns*_3.1_-FLAG plasmid	This study
WT + *hns*_3.2_-FLAG	H111 derivative harboring the pBBR1RS_*hns*_3.2_-FLAG plasmid	This study
Δ*hns*_1.1_ + pBBR1RS	*hns*_1.1_ in-frame deletion mutant harboring the pBBR1RS plasmid	This study
Δ*hns*_1.1_ + *hns*_1.1_-FLAG	*hns*_1.1_ in-frame deletion mutant harboring the pBBR1RS_*hns*_1.1_-FLAG plasmid	This study
Δ*hns*_3.1_ + pBBR1RS	*hns*_3.1_ in-frame deletion mutant harboring the pBBR1RS plasmid	This study
Δ*hns*_3.1_ + *hns*_3.1_-FLAG	*hns*_3.1_ in-frame deletion mutant harboring the pBBR1RS_*hns*_3.1_-FLAG plasmid	This study
WT + pBBR1RS-2	H111 derivative harboring the pBBR1RS-2 plasmid	This study
Δ*hns*_1.2_ + pBBR1RS-2	*hns*_1.2_ in-frame deletion mutant harboring the pBBR1RS-2 plasmid	This study
Δ*hns*_1.2_ + *hns*_1.2_-FLAG	*hns*_1.2_ in-frame deletion mutant harboring the pBBR1RS-2_*hns*_1.2_-FLAG plasmid	This study
*Escherichia coli*		
DH5α	∆*(lacZYA-argF) U169 deoR recA1 endA1 hsdR17 phoA sup144 thi-1 gyrA96 relA1* (j*80 lacZDM15*)	Invitrogen
CC118 λpir	λpir lysogen of CC118 ∆(*ara-leu*) *araD ∆lacX74 galE galK phoA20 thi-1 rpsE rpoB argE (Am) recA1*	(51)
SM10 λpir	*thi thr leu tonA lacY supE recA*::RP4-2-Tc::Mu Km λpir	(52)
Plasmid		
pJET1.2	Linear DNA fragment for blunt ligation	Thermo Fisher Scientific
pGPI-SceI	*ori* R6K, *mob^+^*, I-SceI recognition site, Tp^R^	(53)
pGPI-SceI_*hupA*-FLAG	pGPI derivative harboring the *hupA* coding sequence in frame with the FLAG nucleotide sequence and its upstream region	This study
pGPI-SceI_*hns*_1.1_-FLAG	pGPI derivative harboring the *hns*_1.1_ coding sequence in frame with the FLAG nucleotide sequence and its upstream region	This study
pGPI-SceI_Δ*hns*_1.1_	pGPI derivative harboring a fragment for in-frame deletion of *hns*_1.1_	This study
pGPI-SceI_Δ*hns*_1.2_	pGPI derivative harboring a fragment for in-frame deletion of *hns*_1.2_	This study
pGPI-SceI_Δ*hns*_2_	pGPI derivative harboring a fragment for in-frame deletion of *hns*_2_	This study
pGPI-SceI_Δ*hns*_3.1_	pGPI derivative harboring a fragment for in-frame deletion of *hns*_3.1_	This study
pGPI-SceI_Δ*hns*_3.2_	pGPI derivative harboring a fragment for in-frame deletion of *hns*_3.2_	This study
pDAI-Gm	pDA17 expressing I-SceI nuclease; Gen^R^	(53)
pRK2013	ori *colE1*, RK2 derivative, *mob*^+^, *tra*^+^, Kan^R^	(54)
pBBR1MCS	pBBR1 *ori*V, *mob*^+^, *lacZα*-MCS; broad-host-range cloning vector, Cm^R^	(55)
pBBR1MCS-2	pBBR1 *ori*V, *mob*^+^, *lacZα*-MCS; broad-host-range cloning vector, Kan^R^	(55)
pSC200	Broad-host-range vector, Tp^R^	(56)
pBBR1RS	Replicative plasmid for rhamnose-inducible expression. This plasmid was created by replacing the *lacZ* gene and its promoter in the pBBR1MCS plasmid with the rhamnose system (a rhamnose-inducible promoter divergently positioned to the *rhaSR* operon) from pSC200; Cm^R^	This study
pBBR1RS_*hns*_1.1_-FLAG	pBBR1RS derivative harboring the *hns*_1.1_ coding sequence in frame with the FLAG nucleotide sequence	This study
pBBR1RS_*hns*_1.2_-FLAG	pBBR1RS derivative harboring the *hns*_1.2_ coding sequence in frame with the FLAG nucleotide sequence	This study
pBBR1RS_*hns*_2_-FLAG	pBBR1RS derivative harboring the *hns*_2_ coding sequence in frame with the FLAG nucleotide sequence	This study
pBBR1RS_*hns*_3.1_-FLAG	pBBR1RS derivative harboring the *hns*_3.1_ coding sequence in frame with the FLAG nucleotide sequence	This study
pBBR1RS_*hns*_3.2_-FLAG	pBBR1RS derivative harboring the *hns*_3.2_ coding sequence in frame with the FLAG nucleotide sequence	This study
pBBR1RS-2	Replicative plasmid for rhamnose-inducible expression. This plasmid was created by replacing the *lacZ* gene and its promoter in the pBBR1MCS-2 plasmid with the rhamnose system (a rhamnose-inducible promoter divergently positioned to the *rhaSR* operon) from pSC200; Km^R^	This study
pBBR1RS-2_*hns*_1.2_-FLAG	pBBR1RS-2 derivative harboring the *hns*_1.2_ coding sequence in frame with the FLAG nucleotide sequence	This study

### Genetic manipulation

To create merodiploid strains, DNA fragments encompassing the coding sequence and the upstream intergenic region of *hupA* and *hns*_1.1_ were PCR amplified using primers carrying the FLAG nucleotide sequence immediately before the stop codon of the corresponding gene. The amplicons were cloned into the pJET-1.2 vector using the CloneJET PCR Cloning Kit (Thermo Fisher Scientific) and subcloned in pGPI-SceI, the non-replicative plasmid in *B. cenocepacia*, using restriction enzymes. The pGPI-SceI derivatives were transferred to *B. cenocepacia* H111 by triparental mating, using *E. coli* strains SM10 λpir and DH5α pRK2013 as donor and helper strains, respectively, as previously described (43). In *B. cenocepacia* H111 cells, the transferred plasmids are expected to be integrated into the native locus of *hupA* and *hns*_1.1_ by homologous recombination (Fig. S1A).

Each null mutant strain was created by an in-frame deletion of an *hns* coding sequence using two homologous recombination events. To construct a plasmid for *hns* deletion, two DNA fragments flanking the region to be deleted were separately amplified and then joined by overlap extension PCR. The resulting fragment, whose central part corresponds to a truncated *hns* copy with only a few codons from each end of the original coding sequence, was cloned into pGPI-SceI using restriction enzymes, and the plasmid was transferred to *B. cenocepacia* H111 by triparental mating. The pDAI-Gm vector was then mobilized to *B. cenocepacia* cells carrying a pGPI-SceI derivative to promote the second recombination event. Mutant colonies identified by PCR were grown in LLB agar plates with no antibiotic added and tested for pDAI-Gm curing; those that failed to grow in the presence of gentamicin were isolated as in-frame deletion mutants.

To overexpress an *hns* gene, we constructed replicative plasmids for rhamnose-inducible expression. For these constructions, pBBR1MCS and pBBR1MCS-2 were PCR amplified, except for their regions corresponding to the *lacZ* gene and its promoter. The region of pSC200 encompassing the genes *rhaR* and *rhaS*, along with the rhamnose-inducible promoter, was separately amplified and ligated to the PCR products from pBBR1MCS and pBBR1MCS-2, resulting in the pBBR1RS and pBBR1RS-2 plasmids, respectively. The coding sequence of an *hns* gene was then fused to the DNA fragment amplified from pSC200 by overlap extension PCR using a primer carrying the FLAG nucleotide sequence. The resulting amplicon was used to replace the pSC200-derived region in either pBBR1RS or pBBR1RS-2 using restriction enzymes. Plasmids were transferred to parental cells and in-frame deletion mutants by triparental mating.

The complete lists of plasmids and oligonucleotides used in this study are shown in Table 1 and Table S3, respectively. The inserts cloned into the plasmids were confirmed by sequencing.

### Immunoprecipitation

Cells grown to exponential phase (OD_600nm_ ~0.5) were harvested, washed with 1× TBS (20 mM Tris-Cl pH 7.5, 150 mM NaCl) and 1× TBS supplemented with 10% glycerol, and stored at −80°C. Cell pellets were thawed and resuspended in 1× TBS supplemented with 10% glycerol and Pierce Protease Inhibitor Mini Tablets EDTA-Free (Thermo Fisher Scientific) and disrupted by sonication (2 s on, 10 s off, amplitude 50%, Sonics Vibracell VCX750). The lysate was cleared by centrifugation (21,000 × *g* for 30 min at 4°C), quantified using Bradford Reagent (Merck), and incubated for 16 h at 4°C with anti-FLAG affinity gel (Merck) (10 μL/mg protein) previously equilibrated in 1× TBS supplemented with 10% glycerol. The supernatant was discarded; beads were washed thrice with 1× TBS supplemented with 10% glycerol; and proteins were eluted with 1× SDS loading buffer (half the volume of beads).

### Immunoblotting

Cleared lysates and immunoprecipitation eluates were resolved by SDS-PAGE and transferred to polyvinylidene fluoride membranes (Cytiva). After blocking, membranes were incubated for 16 h at 4°C with anti-FLAG M2 monoclonal antibody (1:1,000; Merck) in 10 mM Tris-Cl, pH 8.0, 150 mM NaCl, 0.02% Tween 20, and 0.03% Triton X-100. The blots were developed using antimouse IgG (whole molecule) conjugated to peroxidase (1:5,000) and ECL Prime Western Blotting Detection Reagent (Cytiva).

### Mass spectrometry

For protein identification, 15 μL of the immunoprecipitation eluate was resolved by SDS-PAGE, and the gel was stained with colloidal Coomassie Brilliant Blue. Bands were isolated and cut into pieces of around 1 mm, and the corresponding proteins were reduced, alkylated, and digested with trypsin. The resulting peptides were eluted, purified by means of ZipTip Pipette Tip C18 resin (Merck), and analyzed by LC-MS/MS.

Mass spectrometric analysis was conducted on an LTQ Orbitrap Velos mass spectrometer (Thermo Fisher Scientific, Bremen, Germany) coupled online to an EASY-nLC 1000 Nanoflow Liquid Chromatography System (Thermo Fisher Scientific). Chromatographic separation was achieved using an Acclaim PepMap NEO analytical column (150 mm × 75 µm, packed with 2 μm C18 particles; Thermo Fisher Scientific) equipped with an Acclaim PepMap 100 C18 trap column (20 mm × 75 µm, 3 μm particles; Thermo Fisher Scientific). Peptides were eluted using a binary gradient at a flow rate of 200 nL/min: first with a linear increase from 2% to 24% solvent B (0.1% formic acid in acetonitrile) over 8 min, followed by an increase from 24% to 80% solvent B over 20 min.

Data were acquired in a data-dependent acquisition mode. Full-scan MS spectra (*m*/*z* 400–1,600) were acquired in the Orbitrap analyzer at a resolution of 30,000. The top 10 most intense precursor ions from each scan cycle were selected for fragmentation via collision-induced dissociation in the linear ion trap. Dynamic exclusion was enabled with a duration of 40 s. The nanoelectrospray ionization source voltage was set to 2.3 kV, and the source temperature was maintained at 250°C. Maximum injection times were set to 100 ms for both FT-MS and ion trap MSⁿ scans.

The raw data files were converted from raw to mzML format using MSConvert (ProteoWizard software, v3.0.23,121). The resulting files were processed using the MSFragger search engine (v19.1) within the FragPipe computational pipeline (v22.0). Database searches were performed against the *B. cenocepacia* J2315 UniProt proteome database (downloaded July 2024). Search parameters were set as follows: precursor and fragment mass tolerances of 10 ppm, peptide length range of 7–50 amino acids, fixed modification of carbamidomethylation on cysteine residues, and variable modification of methionine oxidation. Peptide and protein identifications were filtered using a 1% false discovery rate threshold at both levels.

### Growth analysis

Strains were grown in LLB for 16 h, and the cultures were diluted in fresh medium to OD_600nm_ = 0.03 (*t* = 0). Aliquots for spectrophotometric measurements at OD_600nm_ were taken every hour during a 10-h experiment. To estimate the doubling time for the parental strain and deletion mutants, values for the first 4 h, when growth was considered exponential for all strains, were considered. An exponential trendline was fitted; the equation *y* = *y0*·*e*^kx^ was determined, and the doubling time (*g*) was calculated using *g* = ln(2) / *k*.

### Swarming motility test

Strains were grown in LLB for 16 h, and the cultures were diluted in fresh medium to OD_600nm_ = 0.03, grown to exponential phase (OD_600nm_ ~0.5), and normalized to OD_600nm_ = 0.4. A 3 μL aliquot of each normalized culture was inoculated at the center of a semisolid plate (1% tryptone, 0.5% NaCl, and 0.5% agar). Plates were incubated at room temperature (22°C–25°C) and at 37°C, and the diameter of motility halos was measured every 24 h over 3 days.

### Quantitative biofilm formation analysis

Strains were grown in LLB for 16 h, and the cultures were diluted in fresh medium to OD_600nm_ = 0.03. A volume of 100 μL of each diluted culture was added in triplicate to the dish, which was incubated statically at 37°C for 24 or 48 h. Planktonic cells were discarded, and attached cells were stained with 100 μL 1% crystal violet for 20 min at room temperature. Wells were washed four times with 350 μL Milli-Q water, and stained biofilm was solubilized with 100 μL 80% ethanol and 20% acetone. Absorbance at 570 nm was then determined using a microplate reader (EnSpire Multimode Plate Reader, PerkinElmer).

### Virulence determination

Virulence of *B. cenocepacia* strains was determined using *Galleria mellonella* at the final larval stage. To slow metabolism and prevent pupation, larvae <200 mg were maintained at 16°C. In the meantime, *B. cenocepacia* strains were grown in LLB for 16 h, and the cultures were diluted in fresh medium to OD_600nm_ = 0.03 and grown to exponential phase (OD_600nm_ ~0.5). Larva infection was performed as previously described (57), except for using 0.9% NaCl instead of 1 mM MgSO_4_ as a control and solution to resuspend cells for inoculation.

### RNA sequencing and data processing

Total RNA samples were extracted from cultures at the exponential phase (OD_600nm_ ~0.5) using the Direct-zol RNA Extraction Kit (Zymo Research). Libraries were constructed with 1,000 ng total RNA using the Illumina Stranded Total RNA Prep, Ligation with Ribo-Zero Plus kit, according to the manufacturer’s instructions (Illumina) and quantified by qPCR using the KAPA Library Quantification Kit (Roche). An equimolar pool of the libraries was prepared and sequenced on the NextSeq 2000 (Illumina) using 2 × 100 bp paired-end reads.

RNA-seq reads were processed using a semiautomatic “from raw to coverage” pipeline (58). Briefly, raw data were inspected for quality and size distribution analysis using Rqc95, and Trimmomatic (v0.39) was used for removing adapters and low-quality ends. High-quality sequences were mapped to the *B. cenocepacia* H111 genome (assembly ASM23621v4) using HISAT2 (v2.1.097), retaining only uniquely aligned reads. Bioconductor GenomicAlignments (v1.14.1100) was used to compute the count matrix DESeq2 package (v1.18.1101) (59), and custom R scripts were used for differential gene expression analysis. Genes displaying a log_2_ ratio (mutant/parental) of ≥1.0 or ≤−1.0 and an adjusted *P* value of <0.01 were considered differentially expressed between the strains. The list of orthologous genes between species *B. cenocepacia* H111 and *B. Cenocepacia* J2315 was obtained using the database *Burkholderia* Genome Database (60). The Venn diagram was created using BioVenn (61). Two genes previously reported as belonging to the BDSF stimulon were not considered in this analysis because they could not be found in the genome annotation available at the *Burkholderia* Genome Database.

### Quantitative RT-PCR

Total RNA samples were extracted from cultures at the exponential phase (OD_600nm_ ~0.5) using Trizol reagent and treated with DNase I (Thermo Fisher Scientific). RNA (2.0 μg) was used as template for cDNA synthesis in the presence of 0.2 μg random hexamer primers (Thermo Fisher Scientific), 1 mM dNTPs, and 200 U RevertAid reverse transcriptase (Thermo Fisher Scientific). qPCR was performed with 0.4 μM of each specific primer and SYBR Green Master Mix (Thermo Fisher Scientific). Fluorescence was monitored with a 7500 Fast Real-Time PCR System (Applied Biosystems). Primer sequences were designed using Primer3 (v0.4.0) and are shown in Table S3. Relative expression was calculated using the 2^−ΔΔCT^ method (62), using the *rpoD* gene as an endogenous control for normalization. A gene was considered as differentially expressed when log_2_ fold change is ≤−1 or log_2_ fold change is ≥1, and the adjusted *P* value is ≤0.05.

### Protein sequence alignment

Clustal Omega (63) was used to generate the sequence alignment, and the output was visualized in Jalview 2.11.4.1 (64). Secondary structural elements based on experimentally determined tridimensional structures (PDB: 1HNR e 3NR7) were included in the alignment using ESPrint 3.0 (65).

### Statistical analyses

A one-way ANOVA followed by Dunnett’s test was used to determine whether the deletion mutants and overproducing strains were significantly different from parental cells with respect to growth, motility, biofilm formation, and most of the expression data were obtained by RT-qPCR. A one-way ANOVA followed by Tukey’s test was used for multiple comparisons while analyzing the effect of both cell density and *hns*_1.2_ deletion on gene expression and comparing strains in the complementation experiments. The *G. mellonella* survival curves were generated using Kaplan-Meier and analyzed using log-rank (Mantel-Cox) test. The statistical tests were performed using GraphPad Prism (v9.0.0; GraphPad Software, San Diego, CA, USA), and significance was considered when the *P* value was ≤0.05.

## Data Availability

Raw RNA-seq data are available at the Sequence Read Archive under BioProject accession number PRJNA1353106. The mass spectrometry proteomics data have been deposited to the ProteomeXchange Consortium via the PRIDE partner repository (66) with the data set identifier PXD070299.

## SUPPLEMENTAL MATERIAL

Tables S1 and S2 spectrum.00344-26-s0001.pdf Proteome and transcriptome data.

Figures S1 and S2 spectrum.00344-26-s0002.pdf Figures S1 and S2.

Figures S3 through S7 spectrum.00344-26-s0003.pdf Figures S3 through S7.

Table S3 spectrum.00344-26-s0004.pdf List of oligonucleotides designed and used in this study.

ASM does not own the copyrights to Supplemental Material that may be linked to, or accessed through, an article. The authors have granted ASM a non-exclusive, world-wide license to publish the Supplemental Material files. Please contact the corresponding author directly for reuse.
