# Supplementary material for: A functional interplay of hns genes governs the control of the motile-to-biofilm lifestyle switch in Burkholderia cenocepacia
Source: Microbiol Spectr. 2026 Sep 2;14(10):e00344-26. doi: 10.1128/spectrum.00344-26 (PMC13689386; doi:10.1128/spectrum.00344-26)
Supplement: Supplementary file 1 [file spectrum.00344-26-s0001.pdf]

Table S1. Proteins identified by LC-MS/MS in selected bands

Protein ID[a]	Gene[a]	Protein Description[a]	Length[a]	Coverage[b]	Protein Probability	Top Peptide	Total Peptides	Unique Peptides	Razor Peptides	Total Spectral	Unique Spectral	Razor Spectral	Razor Assigned
**Protein band 1**													
B4E7A4	*hupA*	DNA-binding protein HU-alpha	92	40.22	1.0000	0.9990	4	4	4	11	11	11	1M(15.9949)
B4EDI9	*rpsU*	Small ribosomal subunit protein bS21	70	14.29	1.0000	0.9970	1	1	1	1	1	1	
B4EPN5	BCAS0118	H-NS family DNA-binding protein	109	8.26	1.0000	0.9990	1	1	1	18	18	18	
B4E7I4	BCAL1479	Endoribonuclease L-PSP family protein	117	7.69	0.9409	0.9942	1	1	1	1	1	1	
B4ELY1	BCAM1821	Tautomerase	62	12.90	0.7665	0.9724	1	1	1	1	1	1	
B4EEN9	BCAL2300	Exported protein	110	7.27	0.7660	0.9723	1	1	1	1	1	1	
**Protein band 2**													
B4E7A4	*hupA*	DNA-binding protein HU-alpha	92	41.30	1.0000	0.9990	5	5	5	19	19	19	1M(15.9949)
B4EDI9	*rpsU*	Small ribosomal subunit protein bS21	70	14.29	1.0000	0.9970	1	1	1	4	4	4	
B4EPN5	BCAS0118	H-NS family DNA-binding protein	109	8.26	1.0000	0.9990	1	1	1	1	1	1	
B4EPV7	BCAS0190	H-NS family DNA-binding protein	99	9.09	1.0000	0.9988	1	1	1	1	1	1	3M(15.9949)
B4EE79	BCAL0154	Histone-like nucleoid-structuring (H-NS) protein	97	10.31	0.9997	0.9971	1	1	1	1	1	1	
B4EL50	BCAM0629	Hydrolase	816	0.86	0.9956	0.9801	1	1	1	1	1	1	6M(15.9949)
B4ELS2	BCAM1763	Uncharacterized protein	417	1.92	0.8104	0.9786	1	1	1	1	1	1	6Y(79.9663)
**Protein band 3**													
B4EDJ6	BCAL0122	Histone-like nucleoid-structuring (H-NS) protein	98	14.29	1.0000	0.9990	1	1	1	1	1	1	
B4EPN5	BCAS0118	H-NS family DNA-binding protein	109	14.68	1.0000	0.9990	2	2	2	6	6	6	
B4EPV7	BCAS0190	H-NS family DNA-binding protein	99	18.18	1.0000	0.9988	2	2	2	6	6	6	3M(15.9949)
B4EE79	BCAL0154	Histone-like nucleoid-structuring (H-NS) protein	97	17.53	0.9997	0.9971	2	2	2	4	4	4	
B4EKY0	BCAM2711	H-NS histone family protein	101	7.92	0.9970	0.9784	1	1	1	1	1	1	
B4E9X7	BCAL1796	Saccharopine dehydrogenase	366	2.46	0.9803	0.9981	1	1	1	1	1	1	7M(15.9949)
B4E6X6	BCAL1411	Exported protein	128	8.59	0.9564	0.9958	1	1	1	1	1	1	
**Protein band 4**													
B4EDJ6	BCAL0122	Histone-like nucleoid-structuring (H-NS) protein	98	55.10	1.0000	0.9990	7	7	7	40	40	40	

[a] According to the UniProt
[b] Corresponds to the percentage of the amino acid sequence that is assigned to the peptide(s)

Table S2. Genes differentially expressed in $hns_{1,2}$ mutant cells relative to the parental strain according a RNA-seq-based comparative transcriptome analysis

Locus tag H111[a]	Gene name H111[a]	Old locus tag H111[a]		Locus tag J2315[a]	log_2 fold change[b]	Adjusted *p*-value	Product[a]
**Chr. 1**							
I35_RS00225	I35_RS00225	I35_0046	I35_3176		3.4370	1.6710E-44	hypothetical protein
I35_RS34655	I35_RS34655				1.9452	4.6836E-09	M35 family metallo-endopeptidase
I35_RS00600	I35_RS00600	I35_0119	I35_3096	BCAL0112	-1.2768	2.6855E-07	flagellar protein FliT
I35_RS00605	*fliD*	I35_0120	I35_3095	BCAL0113	-1.3411	2.9316E-07	flagellar filament capping protein FliD
I35_RS00610	I35_RS00610	I35_0121	I35_3094	BCAL0114	-1.5002	3.1604E-09	flagellin
I35_RS00645	*aqpZ*	I35_0128	I35_3086	BCAL0121	2.4353	3.6311E-23	aquaporin Z
I35_RS00650	I35_RS00650	I35_0129	I35_3085	BCAL0122	1.6869	5.3021E-08	H-NS histone family protein
I35_RS00670	*motA*	I35_0133	I35_3080	BCAL0126	-1.0719	5.3931E-07	flagellar motor stator protein MotA
I35_RS00675	*motB*	I35_0134	I35_3079	BCAL0127	-1.2273	7.1839E-06	flagellar motor protein MotB
I35_RS00680	I35_RS00680	I35_0135	I35_3078	BCAL0128	-1.0911	1.6100E-06	response regulator
I35_RS00690	*cheW*	I35_0137	I35_0297	BCAL0130	-1.0924	3.3431E-05	chemotaxis protein CheW
I35_RS00700	I35_RS00700	I35_0139	I35_5070	BCAL0132	-1.3621	6.5926E-07	CheR family methyltransferase
I35_RS00705	*cheD*	I35_0140	I35_5069	BCAL0133	-1.2292	7.7475E-04	chemoreceptor glutamine deamidase CheD
I35_RS00710	I35_RS00710	I35_0141	I35_5068	BCAL0134	-1.3751	2.1958E-08	chemotaxis-specific protein-glutamate methyltransferase CheB
I35_RS00720	*cheZ*	I35_0143	I35_5066	BCAL0136	-1.0445	7.5734E-05	protein phosphatase CheZ
I35_RS00810	I35_RS00810	I35_0161	I35_5046	BCAL0154	-3.8707	9.1037E-64	H-NS histone family protein
I35_RS00815	I35_RS00815	I35_0162	I35_5045	BCAL0155	2.5462	6.3275E-24	cation diffusion facilitator family transporter
I35_RS01800	I35_RS01800	I35_0349	I35_0368	BCAL0358	5.4998	8.4659E-120	M1 family metallopeptidase
I35_RS01805	I35_RS01805	I35_0350	I35_0367	BCAL0359	1.4418	5.3668E-04	LEA type 2 family protein
I35_RS02665	I35_RS02665	I35_0513	I35_3884	BCAL3362	-1.2346	2.0590E-04	FAD-dependent oxidoreductase
I35_RS02705	I35_RS02705	I35_0521	I35_5206	BCAL3353	1.8681	3.1028E-06	autotransporter outer membrane beta-barrel domain-containing protein
I35_RS03025	*hmpA*	I35_0589	I35_5138	BCAL3285	4.3886	1.4324E-84	NO-inducible flavohemoprotein
I35_RS03195	I35_RS03195			BCAL3244	1.2747	1.4910E-11	glycosyltransferase family 1 protein
I35_RS03200	I35_RS03200	I35_0629	I35_5098	BCAL3243	1.0254	4.7205E-09	polysaccharide biosynthesis/export family protein
I35_RS03205	I35_RS03205				1.3968	8.2928E-17	glycosyltransferase family 1 protein
I35_RS36625	I35_RS36625				1.5420	5.4056E-20	hypothetical protein
I35_RS34805	I35_RS34805	I35_0630	I35_5097		2.0144	1.1520E-30	FkbM family methyltransferase
I35_RS03210	I35_RS03210	I35_0631	I35_5096		1.9986	2.3801E-39	glycosyltransferase
I35_RS34810	I35_RS34810				2.0598	9.7981E-27	ABC transporter ATP-binding protein
I35_RS03220	I35_RS03220	I35_0632	I35_5095	BCAL3242	1.1032	1.1348E-09	hypothetical protein
I35_RS03225	I35_RS03225	I35_0633	I35_5093		1.2279	5.3472E-14	capsular biosynthesis protein
I35_RS03230	I35_RS03230			BCAL3222	1.2020	1.8499E-07	SDR family NAD(P)-dependent oxidoreductase
I35_RS03260	*cysC*	I35_0640	I35_5086	BCAL3216	1.1774	9.2409E-14	adenylyl-sulfate kinase
I35_RS03440	*bluB*	I35_0677	I35_3859	BCAL3180	1.3169	5.7306E-04	5,6-dimethylbenzimidazole synthase
I35_RS03445	I35_RS03445	I35_0678	I35_3858	BCAL3179	1.6818	5.0457E-13	2-hydroxyacid dehydrogenase
I35_RS03450	I35_RS03450	I35_0679	I35_3857	BCAL3178	1.7018	2.9277E-14	LysR family transcriptional regulator
I35_RS03520	I35_RS03520	I35_0698	I35_3838	BCAL3164	5.4255	1.7249E-72	peptidase S1
I35_RS03525	I35_RS03525	I35_0700	I35_3836	BCAL3163	1.6175	1.5234E-03	nucleotidyltransferase domain-containing protein
I35_RS03570	I35_RS03570	I35_0709	I35_3827	BCAS0321a	2.9168	9.1609E-03	hypothetical protein
I35_RS04160	*ugpC*	I35_0830	I35_3705	BCAL3038	-1.0730	1.6737E-03	sn-glycerol-3-phosphate ABC transporter ATP-binding protein UgpC
I35_RS04350	I35_RS04350	I35_0863	I35_3667	BCAL3006	1.4929	3.8704E-21	cold-shock protein
I35_RS04355	I35_RS04355	I35_0864	I35_3666	BCAL3005	1.1211	5.3021E-08	exonuclease domain-containing protein
I35_RS04370	I35_RS04370	I35_0868	I35_3662	BCAL3002	-1.9180	2.3081E-06	DUF2946 domain-containing protein
I35_RS04375	I35_RS04375	I35_0869	I35_3661	BCAL3001	-2.3400	6.8807E-09	TonB-dependent receptor
I35_RS04380	I35_RS04380	I35_0870	I35_3660	BCAL3000	-1.8322	1.2776E-03	sialidase family protein
I35_RS04815	I35_RS04815	I35_0963	I35_3566	BCAL2904	-1.3032	7.0512E-08	DUF4088 family protein
I35_RS05975	I35_RS05975	I35_1205	I35_5298	BCAL1291	1.0600	5.1559E-04	DUF3592 domain-containing protein

I35_RS05985	*tssF*	I35_1207	I35_5297	BCAL1293	1.4341	3.2958E-08	type VI secretion system baseplate subunit TssF
I35_RS05990	*tssI*	I35_1208	I35_5296	BCAL1294	1.6932	1.8195E-19	type VI secretion system tip protein VgrG
I35_RS34580	I35_RS34580	I35_1209			1.3375	1.6013E-07	DUF2169 domain-containing protein
I35_RS06000	I35_RS06000	I35_1210	I35_5295	BCAL1296	1.1226	8.6051E-09	PAAR-like domain-containing protein
I35_RS06005	I35_RS06005	I35_1211	I35_5294	BCAL1297	1.3062	5.1132E-11	ankyrin repeat domain-containing protein
I35_RS37520	I35_RS37520				1.0456	7.1253E-08	DNA/RNA non-specific endonuclease
I35_RS34940	I35_RS34940				1.2984	1.9875E-12	immunity protein YezG family protein
I35_RS36680	I35_RS36680				1.9074	4.6645E-29	hypothetical protein
I35_RS34945	I35_RS34945				1.8613	3.2580E-15	SMI1/KNR4 family protein
I35_RS36685	I35_RS36685				1.8072	4.7225E-17	hypothetical protein
I35_RS34960	I35_RS34960				1.5066	5.4299E-08	hypothetical protein
I35_RS06035	I35_RS06035	I35_1213	I35_5292	BCAL1318	1.2585	7.3540E-03	nitrate regulatory protein
I35_RS06220	I35_RS06220	I35_1250	I35_5248	BCAL1355	1.1493	2.9316E-07	type VI secretion system Vgr family protein
I35_RS06255	I35_RS06255	I35_1257	I35_5241	BCAL1363	1.1115	5.0851E-04	DUF4123 domain-containing protein
I35_RS06280	I35_RS06280	I35_1262	I35_5236	BCAL1368	-1.0908	2.0396E-04	porin
I35_RS06660	I35_RS06660	I35_1340	I35_5560	BCAL1442	1.1167	6.7175E-07	hypothetical protein
I35_RS06715	I35_RS06715	I35_1352	I35_5547	BCAL1453	-1.6400	7.9507E-03	HlyD family secretion protein
I35_RS06725	I35_RS06725	I35_1354	I35_5545	BCAL1455	-1.5107	4.3698E-03	FUSC family protein
I35_RS06735	I35_RS06735	I35_1356	I35_5543	BCAL1457	-1.1235	2.0233E-05	LysR family transcriptional regulator
I35_RS07055	I35_RS07055	I35_1419	I35_1711	BCAL1522	1.1561	2.6102E-03	ShlB/FhaC/HecB family hemolysin secretion/activation protein
I35_RS07065	I35_RS07065	I35_1421	I35_1709		3.5572	1.7803E-14	collagen-like triple helix repeat-containing protein
I35_RS07070	I35_RS07070	I35_1422	I35_1708	BCAL1525	1.7936	2.8813E-18	Flp family type IVb pilin
I35_RS07075	I35_RS07075	I35_1423	I35_1707	BCAL1525a	2.0216	2.6148E-20	A24 family peptidase
I35_RS07080	I35_RS07080	I35_1424	I35_1706	BCAL1526	2.2558	4.1756E-28	TadE family protein
I35_RS07085	*cpaB*	I35_1425	I35_1705	BCAL1527	2.1952	1.4233E-21	Flp pilus assembly protein CpaB
I35_RS07090	I35_RS07090	I35_1426	I35_1704	BCAL1528	2.0612	3.2466E-30	type II and III secretion system protein family protein
I35_RS07095	I35_RS07095	I35_1427	I35_1703	BCAL1529	2.0036	1.0656E-16	fimbrial protein
I35_RS07100	I35_RS07100	I35_1428	I35_1702	BCAL1530	2.2625	9.5878E-29	CpaF family protein
I35_RS07105	I35_RS07105	I35_1429	I35_1701	BCAL1531	2.5481	7.7586E-15	type II secretion system F family protein
I35_RS07110	I35_RS07110	I35_1430	I35_1700	BCAL1532	2.4975	5.7548E-20	type II secretion system F family protein
I35_RS07115	I35_RS07115	I35_1431	I35_1699	BCAL1533	1.9944	1.5170E-21	hypothetical protein
I35_RS07120	I35_RS07120	I35_1432	I35_1698	BCAL1534	2.0508	7.2230E-04	DUF3613 domain-containing protein
I35_RS07125	I35_RS07125	I35_1433	I35_1697	BCAL1535	2.0069	8.8451E-20	TadG family pilus assembly protein
I35_RS07130	I35_RS07130	I35_1434	I35_1696	BCAL1536	2.1285	8.0569E-23	sigma-54 dependent transcriptional regulator
I35_RS07385	I35_RS07385	I35_1480	I35_1646		1.7109	1.6246E-03	hypothetical protein
I35_RS07405	I35_RS07405	I35_1484	I35_1642		1.9756	6.7698E-05	RNA-directed DNA polymerase
I35_RS07410	I35_RS07410	I35_1485	I35_1641		1.6439	1.0789E-03	four helix bundle protein
I35_RS07415	I35_RS07415	I35_1486	I35_1640		2.5402	1.8120E-07	DUF1566 domain-containing protein
I35_RS07420	I35_RS07420	I35_1487	I35_1639		2.2977	1.3344E-08	hypothetical protein
I35_RS07425	I35_RS07425	I35_1488	I35_1638		2.3576	1.3135E-03	hypothetical protein
I35_RS07440	I35_RS07440	I35_1491	I35_1635		1.7339	6.0422E-05	hypothetical protein
I35_RS37860	I35_RS37860	I35_1492	I35_1634		3.2117	4.7276E-04	hypothetical protein
I35_RS07485	I35_RS07485	I35_1499	I35_1627		2.1553	2.8041E-03	hypothetical protein
I35_RS35060	I35_RS35060	I35_1500	I35_1626		2.9161	3.3233E-05	helix-turn-helix domain-containing protein
I35_RS07540	I35_RS07540	I35_1510	I35_1616		1.5994	6.6197E-03	phage terminase large subunit family protein
I35_RS07550	I35_RS07550	I35_1511	I35_1615		1.9335	8.2591E-03	phage portal protein
I35_RS07565	I35_RS07565	I35_1514	I35_1612		2.7907	1.8514E-03	head decoration protein
I35_RS07570	I35_RS07570	I35_1515	I35_1611		3.1905	2.1641E-04	major capsid protein
I35_RS07595	I35_RS07595	I35_1520	I35_1606		2.9030	3.0720E-05	phage tail sheath subtilisin-like domain-containing protein
I35_RS07700	I35_RS07700	I35_1537	I35_1586	BCAL1627	1.4716	1.7608E-05	GNAT family N-acetyltransferase
I35_RS07710	I35_RS07710	I35_1539	I35_1584	BCAL1629	1.6321	1.2946E-03	hypothetical protein
I35_RS07915	I35_RS07915	I35_1581	I35_7389		1.5087	1.0028E-10	hemagglutinin repeat-containing protein
I35_RS35100	I35_RS35100				1.7095	1.4935E-12	contact-dependent growth inhibition system immunity protein

I35_RS07925	I35_RS07925	I35_1582	I35_7388		1.4626	1.1682E-12	ShlB/FhaC/HecB family hemolysin secretion/activation protein
I35_RS07955	I35_RS07955	I35_1588	I35_7381	BCAL1677	3.1957	4.6138E-42	fimbrial protein
I35_RS07960	I35_RS07960	I35_1589	I35_7379	BCAL1678	3.2059	1.5572E-49	fimbria/pilus outer membrane usher protein
I35_RS07965	I35_RS07965	I35_1590	I35_7378	BCAL1679	3.1746	3.2528E-38	molecular chaperone
I35_RS07970	I35_RS07970	I35_1591	I35_7377	BCAL1680	3.0035	3.3598E-40	fimbrial protein
I35_RS07975	I35_RS07975	I35_1592	I35_7376	BCAL1681	3.0773	1.6425E-43	hypothetical protein
I35_RS08545	I35_RS08545	I35_1710	I35_4668	BCAL1795	1.1047	3.6056E-03	ATP-binding protein
I35_RS08705	I35_RS08705	I35_1743	I35_4634	BCAL1826	2.5874	1.2100E-36	DUF1120 domain-containing protein
I35_RS08710	I35_RS08710	I35_1744	I35_4633	BCAL1827	2.2637	2.4438E-13	fimbria/pilus chaperone family protein
I35_RS08715	I35_RS08715	I35_1745	I35_4632	BCAL1828	2.9658	5.0713E-12	fimbria/pilus outer membrane usher protein
I35_RS09165	I35_RS09165	I35_1837	I35_3345	BCAL1917	1.2223	9.6980E-07	helix-hairpin-helix domain-containing protein
I35_RS09170	I35_RS09170	I35_1838	I35_3344	BCAL1918	1.3629	4.6234E-08	YidB family protein
I35_RS09185	I35_RS09185	I35_1841	I35_3341	BCAL1921	2.4156	4.7005E-11	group III truncated hemoglobin
I35_RS09340	*lysM*	I35_1872	I35_3309	BCAL1952	1.3851	4.5301E-10	peptidoglycan-binding protein LysM
I35_RS09450	I35_RS09450	I35_1894	I35_3286	BCAL1972	1.3336	3.1129E-03	hypothetical protein
I35_RS09455	I35_RS09455	I35_1895	I35_3285	BCAL1973	1.3815	5.6920E-04	RidA family protein
I35_RS09460	I35_RS09460	I35_1896	I35_3284	BCAL1974	5.1577	2.3701E-90	hypothetical protein
I35_RS11015	I35_RS11015	I35_2201	I35_5596	BCAL2272	1.1780	5.2197E-09	hypothetical protein
I35_RS11020	I35_RS11020	I35_2202	I35_5595	BCAL2273/BCAL2275	1.1021	4.1309E-04	PAAR domain-containing protein
I35_RS11025	I35_RS11025	I35_2203	I35_5594	BCAL2276	1.0135	4.5312E-09	hypothetical protein
I35_RS11130	I35_RS11130	I35_2223	I35_4020	BCAL2297	1.1185	8.7188E-06	hemin uptake protein HemP
I35_RS11410	I35_RS11410	I35_2276	I35_4355	BCAL2352	2.6631	2.1975E-32	carbonic anhydrase
I35_RS11415	I35_RS11415	I35_2277	I35_4356	BCAL2353	2.8175	2.8236E-19	SulP family inorganic anion transporter
I35_RS37405	I35_RS37405	I35_2424	I35_4504		1.4813	9.8183E-03	hypothetical protein
I35_RS12240	I35_RS12240	I35_2447	I35_4526		1.1797	4.8759E-06	hypothetical protein
I35_RS12455	I35_RS12455	I35_2488	I35_4571	BCAL2627	1.3880	6.8064E-03	alpha/beta fold hydrolase
I35_RS12735	I35_RS12735	I35_2544	I35_2878	BCAL2684	-1.3374	1.6691E-03	DUF934 domain-containing protein
I35_RS12740	I35_RS12740	I35_2545	I35_2879	BCAL2685	-1.1291	7.1090E-05	nitrite/sulfite reductase
I35_RS13235	I35_RS13235	I35_2643	I35_3491	BCAL2786	1.9257	1.4686E-08	selenium-binding protein SBP56-related protein
I35_RS14195	I35_RS14195	I35_2837	I35_6752	BCAL0834	5.5131	3.2438E-05	hypothetical protein
I35_RS14200	*phbB*	I35_2838	I35_6753	BCAL0833	5.4229	3.0578E-59	acetoacetyl-CoA reductase
I35_RS14205	I35_RS14205	I35_2839	I35_8013	BCAL0832	4.4362	2.8242E-113	acetyl-CoA C-acetyltransferase
I35_RS14210	phaP	I35_2840	I35_7219	BCAL0831	4.2006	4.9504E-109	TIGR01841 family phasin
I35_RS14520	I35_RS14520	I35_2905	I35_7289	BCAL0762	-1.2487	1.0427E-03	methyl-accepting chemotaxis protein
I35_RS14995	*bioA*	I35_2998	I35_1870	BCAL0667	-1.1847	5.9432E-05	adenosylmethionine--8-amino-7-oxononanoate transaminase
I35_RS15205	I35_RS15205	I35_3041	I35_1915	BCAL0625	1.7173	1.1831E-12	LysR family transcriptional regulator
I35_RS15380	I35_RS15380	I35_3077	I35_1954	BCAL0589	1.9002	6.8675E-10	DUF799 domain-containing protein
I35_RS15385	I35_RS15385	I35_3078	I35_1955	BCAL0588	1.7732	5.6583E-08	DUF4810 domain-containing protein
I35_RS15390	I35_RS15390	I35_3079	I35_1956	BCAL0587	1.9602	1.6987E-13	CsgG/HfaB family protein
I35_RS15440	*flgL*	I35_3089	I35_1966	BCAL0577	-1.4369	1.2726E-11	flagellar hook-associated protein FlgL
I35_RS15445	*flgK*	I35_3090	I35_1967	BCAL0576	-1.4892	2.4525E-13	flagellar hook-associated protein FlgK
I35_RS15675	I35_RS15675	I35_3137	I35_2017	BCAL0528	-1.0294	7.0828E-04	flagellar protein FliT
I35_RS16235	I35_RS16235	I35_3251	I35_6791		1.8677	1.7901E-25	type I restriction endonuclease subunit R
I35_RS16240	I35_RS16240	I35_3252	I35_6792		1.8529	1.5732E-21	hypothetical protein
**Chr. 2**							
I35_RS16620	*tdh*	I35_4011	I35_3044	BCAM0011	-1.2895	1.5311E-04	L-threonine 3-dehydrogenase
I35_RS36855	I35_RS36855				2.1709	3.4802E-12	hypothetical protein
I35_RS16705	I35_RS16705	I35_4028	I35_0684	BCAM0028	3.0601	1.8134E-20	FHA domain-containing protein
I35_RS16710	I35_RS16710	I35_4029	I35_0683	BCAM0029	1.2895	2.4069E-04	TOMM system kinase/cyclase fusion protein
I35_RS16715	I35_RS16715	I35_4030	I35_0682	BCAM0030	2.2907	3.9369E-03	BMA_0021/BMA_0022 family TOMM bacteriocin
I35_RS16895	I35_RS16895	I35_4065	I35_0645	BCAM0065	2.3352	1.8774E-09	LysE family translocator
I35_RS35550	I35_RS35550				2.3363	1.0643E-41	hypothetical protein
I35_RS35555	I35_RS35555				1.9698	4.9021E-27	serine/threonine-protein kinase

I35_RS37280	I35_RS37280					1.5152	7.0509E-14	hypothetical protein
I35_RS16905	I35_RS16905	I35_4067	I35_0643			2.9925	1.1511E-12	hypothetical protein
I35_RS16910	I35_RS16910					3.1116	6.1327E-20	helix-turn-helix transcriptional regulator
I35_RS37630	I35_RS37630					3.9083	1.2176E-09	TnsA endonuclease N-terminal domain-containing protein
I35_RS16920	I35_RS16920	I35_4069	I35_0641	BCAL1592		2.6936	1.4925E-07	Mu transposase C-terminal domain-containing protein
I35_RS16940	I35_RS16940	I35_4074	I35_8024			1.2664	1.3719E-06	hypothetical protein
I35_RS16945	I35_RS16945					2.0058	5.1730E-06	hypothetical protein
I35_RS36885	I35_RS36885	I35_4081	I35_8032			1.0157	1.4312E-06	hypothetical protein
I35_RS17330	*tssH*	I35_4152	I35_0564			2.2824	8.5972E-05	type VI secretion system ATPase TssH
I35_RS17335	I35_RS17335	I35_4153	I35_0563			2.0402	1.2811E-07	Hcp family type VI secretion system effector
I35_RS17340	I35_RS17340	I35_4154	I35_0562			1.6414	3.6084E-03	OmpA family protein
I35_RS17350	*tssK*	I35_4156	I35_0560			2.0466	8.3945E-06	type VI secretion system baseplate subunit TssK
I35_RS17355	*tssB*	I35_4157	I35_0559			2.4897	1.6746E-04	type VI secretion system contractile sheath small subunit
I35_RS17360	*tssC*	I35_4158	I35_0558			2.1890	9.4893E-07	type VI secretion system contractile sheath large subunit
I35_RS17365	*vgrG*	I35_4159	I35_0557			1.5258	1.6737E-03	type VI secretion system Vgr family protein
I35_RS17445	*mqo*	I35_4174	I35_0540	BCAM0175		-1.1230	1.7557E-04	malate dehydrogenase (quinone)
I35_RS17480	I35_RS17480	I35_4181	I35_0533			4.7698	4.9091E-10	GyrI-like domain-containing protein
I35_RS17485	I35_RS17485	I35_4182	I35_0532	BCAM0184		5.3201	3.7014E-38	fucose-binding lectin II
I35_RS17490	I35_RS17490	I35_4183	I35_0531	BCAM0185		5.9088	5.1564E-69	fucose-binding lectin II
I35_RS17495	I35_RS17495	I35_4184	I35_0530	BCAM0186		5.5706	1.9637E-25	fucose-binding lectin II
I35_RS17505	I35_RS17505	I35_4186	I35_0528	BCAM0188		2.2308	5.3297E-10	autoinducer binding domain-containing protein
I35_RS17510	I35_RS17510	I35_4187	I35_0527	BCAM0189		3.0419	3.5661E-19	helix-turn-helix transcriptional regulator
I35_RS17515	I35_RS17515	I35_4188	I35_0526	BCAM0190		5.1214	2.2226E-16	aspartate aminotransferase family protein
I35_RS17520	I35_RS17520	I35_4189	I35_0525	BCAM0191		4.7397	6.2602E-12	condensation domain-containing protein
I35_RS17525	I35_RS17525	I35_4191	I35_0523	BCAM0192		6.1283	6.2263E-12	DUF3050 domain-containing protein
I35_RS17530	I35_RS17530	I35_4192	I35_0522	BCAM0193		7.0437	4.4806E-05	hypothetical protein
I35_RS17535	I35_RS17535	I35_4193	I35_0521	BCAM0194		5.6990	2.6929E-12	diiron oxygenase
I35_RS17540	I35_RS17540	I35_4194	I35_0520	BCAM0195		5.6660	8.3719E-11	amino acid adenylation domain-containing protein
I35_RS17545	I35_RS17545	I35_4195	I35_0519	BCAM0196		5.5732	1.7716E-09	SRPBCC family protein
I35_RS17560	I35_RS17560	I35_4198	I35_0516	BCAM0199		5.4975	7.3341E-14	TolC family protein
I35_RS17565	I35_RS17565	I35_4199	I35_0515	BCAM0200		4.6460	2.1314E-11	HlyD family secretion protein
I35_RS17570	I35_RS17570	I35_4200	I35_0514	BCAM0201		4.3478	8.4164E-10	DHA2 family efflux MFS transporter permease subunit
I35_RS19215	I35_RS19215	I35_4529	I35_2219	BCAM0633		2.9132	4.2434E-15	hypothetical protein
I35_RS37895	I35_RS37895	I35_4530	I35_2218			3.0143	1.3395E-16	hypothetical protein
I35_RS19520	I35_RS19520	I35_4592	I35_2151	BCAM0694		2.0497	2.2595E-03	isoprenylcysteine carboxylmethyltransferase family protein
I35_RS36970	I35_RS36970	I35_4593	I35_2150			2.1370	1.1439E-08	collagen-like triple helix repeat-containing protein
I35_RS35700	I35_RS35700					2.6555	4.7608E-21	OmpA family protein
I35_RS19595	I35_RS19595	I35_4608	I35_2827	BCAM0710		2.4753	1.5643E-13	M35 family metallo-endopeptidase
I35_RS19600	I35_RS19600	I35_4609	I35_2826	BCAM0711		3.2322	1.6167E-07	TolC family protein
I35_RS19605	I35_RS19605	I35_4610	I35_2825	BCAM0712		3.4638	7.4143E-04	efflux RND transporter periplasmic adaptor subunit
I35_RS19610	I35_RS19610	I35_4611	I35_2824	BCAM0713		2.6228	1.5754E-05	CusA/CzcA family heavy metal efflux RND transporter
I35_RS19920	I35_RS19920	I35_4674	I35_2755	BCAM0776		-1.3222	6.8684E-05	cyclic nucleotide-binding domain-containing protein
I35_RS19930	I35_RS19930	I35_4676	I35_2753	BCAM0778		-1.6006	6.7091E-04	OmpA family protein
I35_RS19935	I35_RS19935	I35_4677	I35_2752	BCAM0779		-1.3608	1.4099E-04	methyl-accepting chemotaxis protein
I35_RS19940	I35_RS19940	I35_4679	I35_2750	BCAM0781		2.5790	1.3557E-20	hypothetical protein
I35_RS20120	I35_RS20120	I35_4717	I35_2712	BCAM0819		1.1459	5.4489E-03	Na+/H+ antiporter
I35_RS20125	I35_RS20125	I35_4718	I35_2711	BCAM0820		1.3092	9.4552E-09	hybrid sensor histidine kinase/response regulator
I35_RS20130	I35_RS20130	I35_4719	I35_2709	BCAM0821		1.3633	2.6971E-10	methyl-accepting chemotaxis protein
I35_RS20135	I35_RS20135	I35_4720	I35_2708	BCAM0822		1.5345	9.9112E-03	chemotaxis protein CheW
I35_RS20145	I35_RS20145	I35_4722	I35_4828	BCAM0824		1.6916	1.1975E-04	chemotaxis protein CheW
I35_RS20150	I35_RS20150	I35_4723	I35_4827	BCAM0825		1.0567	4.3013E-03	response regulator
I35_RS20255	I35_RS20255	I35_4747	I35_4803	BCAM0835		2.5958	3.6646E-23	helix-turn-helix domain-containing protein
I35_RS20260	I35_RS20260	I35_4748	I35_4802	BCAM0836		1.0070	2.6069E-03	Nramp family divalent metal transporter

I35_RS20340	I35_RS20340	I35_4766	I35_4785	BCAM0853	2.1987	1.4590E-14	DUF6566 family protein
I35_RS20345	I35_RS20345	I35_4767	I35_4784	BCAM0854	5.2831	4.2269E-95	mannose-1-phosphate guanylyltransferase/mannose-6-phosphate isomerase
I35_RS20350	I35_RS20350	I35_4768	I35_4782		6.3690	6.6991E-47	undecaprenyl-phosphate glucose phosphotransferase
I35_RS20355	I35_RS20355	I35_4769	I35_4781	BCAM0855	5.7893	2.7534E-47	UDP-glucose/GDP-mannose dehydrogenase family protein
I35_RS20360	I35_RS20360	I35_4770	I35_4780	BCAM0857	6.3570	1.6579E-14	low molecular weight protein-tyrosine-phosphatase
I35_RS20365	I35_RS20365	I35_4771	I35_4779	BCAM0858	5.6150	3.9978E-38	polysaccharide biosynthesis/export family protein
I35_RS20370	I35_RS20370	I35_4772	I35_4778	BCAM0859	5.4956	7.7592E-60	polysaccharide biosynthesis tyrosine autokinase
I35_RS20375	I35_RS20375	I35_4773	I35_4777	BCAM0860	4.8100	3.2254E-25	glycosyltransferase family 2 protein
I35_RS20380	I35_RS20380	I35_4774	I35_4776	BCAM0861	6.3010	3.3401E-14	glycosyltransferase family 4 protein
I35_RS20385	I35_RS20385	I35_4775		BCAM0862	5.4081	5.1305E-20	hypothetical protein
I35_RS20390	I35_RS20390	I35_4776	I35_4774	BCAM0863	5.2516	8.6183E-21	glycosyltransferase family 4 protein
I35_RS20395	I35_RS20395	I35_4777	I35_4773	BCAM0864	5.1822	2.7559E-26	glycosyltransferase family 4 protein
I35_RS35765	I35_RS35765	I35_4815	I35_8034		1.9082	3.7407E-10	hypothetical protein
I35_RS20580	I35_RS20580				1.3010	9.6762E-04	DUF5677 domain-containing protein
I35_RS21090	I35_RS21090	I35_4922	I35_2574	BCAM1003	2.5551	3.4861E-21	NAD-dependent epimerase/dehydratase family protein
I35_RS21095	*gmd*	I35_4923	I35_2573	BCAM1004	5.8664	1.5122E-83	GDP-mannose 4,6-dehydratase
I35_RS21100	I35_RS21100	I35_4924	I35_2572	BCAM1005	5.9930	9.7769E-32	acyltransferase
I35_RS21105	I35_RS21105	I35_4925	I35_2571	BCAM1006	5.2800	6.1777E-37	hypothetical protein
I35_RS21110	I35_RS21110	I35_4926	I35_2570	BCAM1007	4.6976	5.3824E-16	flippase
I35_RS21115	I35_RS21115	I35_4927	I35_2569	BCAM1008	4.8460	1.3037E-38	glycosyltransferase family 4 protein
I35_RS21120	I35_RS21120	I35_4928	I35_2567	BCAM1009	4.1617	7.1136E-24	acyltransferase
I35_RS37450	I35_RS37450				6.9311	4.5033E-34	hypothetical protein
I35_RS21125	*galU*	I35_4929	I35_2565	BCAM1010	5.4417	5.8383E-106	UTP--glucose-1-phosphate uridylyltransferase GalU
I35_RS21645	I35_RS21645	I35_5034	I35_1396	BCAM1187	-3.8952	5.0446E-41	TonB-dependent receptor
I35_RS21730	*alr*	I35_5051	I35_1379	BCAM1204	1.0567	2.3448E-03	alanine racemase
I35_RS21915	I35_RS21915	I35_5089	I35_1339	BCAM1241	3.7493	1.1074E-06	acyltransferase
I35_RS22295	I35_RS22295	I35_5165	I35_1259	BCAM1316	2.5244	1.6167E-07	TolC family protein
I35_RS37295	I35_RS37295	I35_5166	I35_1258		2.6786	8.0779E-10	hypothetical protein
I35_RS22300	I35_RS22300	I35_5167	I35_1257	BCAM1316b	5.5674	1.6617E-05	hypothetical protein
I35_RS22430	I35_RS22430	I35_5195	I35_1227	BCAM1344	2.8603	5.7564E-03	DUF1839 family protein
I35_RS22440	I35_RS22440	I35_5197	I35_1225	BCAM1346	4.6038	2.5301E-03	acyl-CoA dehydrogenase family protein
I35_RS22445	I35_RS22445	I35_5198	I35_1224	BCAM1347	3.1616	3.0870E-03	acyl carrier protein
I35_RS22450	I35_RS22450	I35_5199	I35_1223	BCAM1348	4.6425	4.2294E-13	Crp/Fnr family transcriptional regulator
I35_RS22525	I35_RS22525	I35_5215	I35_1207	BCAM1363	1.4065	2.4588E-06	DUF3564 family protein
I35_RS22770	I35_RS22770	I35_5266	I35_1152	BCAM1412	4.2682	1.2461E-29	inclusion body family protein
I35_RS22775	I35_RS22775	I35_5267	I35_1151	BCAM1413a	4.0112	1.2297E-68	AidA/PixA family protein
I35_RS22830	I35_RS22830	I35_5278	I35_1140	BCAM1424	-1.2551	5.4785E-03	methyl-accepting chemotaxis protein
I35_RS23120	I35_RS23120	I35_5337	I35_1079	BCAM1483	1.1933	1.2242E-05	helix-turn-helix domain-containing protein
I35_RS23125	I35_RS23125	I35_5338	I35_1078	BCAM1484	1.0247	1.8291E-04	response regulator transcription factor
I35_RS23270	I35_RS23270	I35_5368	I35_1047	BCAM1514	1.2117	9.8878E-03	MipA/OmpV family protein
I35_RS24250	I35_RS24250	I35_5563	I35_0831		2.7064	8.2591E-03	fimbria/pilus periplasmic chaperone
I35_RS24255	I35_RS24255	I35_5564	I35_0830		2.3533	5.8651E-11	fimbrial protein
I35_RS24260	I35_RS24260	I35_5565	I35_0829		2.9420	1.0691E-03	response regulator transcription factor
I35_RS24265	I35_RS24265	I35_5566	I35_0828	BCAM1695	3.0670	1.0120E-10	trypsin-like peptidase domain-containing protein
I35_RS24270	I35_RS24270	I35_5567	I35_0827	BCAM1696	3.6872	1.9915E-26	hypothetical protein
I35_RS24275	I35_RS24275	I35_5568	I35_0826	BCAM1697	3.1774	1.9393E-14	type II CAAX endopeptidase family protein
I35_RS24420	I35_RS24420	I35_5596	I35_0795	BCAM1726	1.2626	2.1857E-09	hypothetical protein
I35_RS24700	I35_RS24700	I35_5655	I35_4294	BCAM1777A	-1.7488	7.7491E-05	hypothetical protein
I35_RS24830	I35_RS24830	I35_5683	I35_4266	BCAM1804	-1.1116	1.1554E-05	methyl-accepting chemotaxis protein
I35_RS24865	I35_RS24865	I35_5690	I35_4259	BCAM1811	-1.2099	3.8812E-04	hypothetical protein
I35_RS25335	*tssF*	I35_5783	I35_4162		1.6299	3.5113E-03	type VI secretion system baseplate subunit TssF
I35_RS37745	I35_RS37745	I35_5784	I35_4161		2.1888	5.5188E-05	type VI secretion IcmF C-terminal domain-containing protein
I35_RS25340	I35_RS25340	I35_5785	I35_4160		2.4896	3.0351E-16	ImcF-related family protein

I35_RS25345	I35_RS25345				2.5819	2.3636E-18	hypothetical protein
I35_RS37020	I35_RS37020				2.3473	1.3897E-13	hypothetical protein
I35_RS36060	I35_RS36060	I35_5786	I35_4159		1.1394	1.9861E-03	hypothetical protein
I35_RS25625	I35_RS25625	I35_5842	I35_2976	BCAM2007	-2.3960	4.2434E-15	TonB-dependent siderophore receptor
I35_RS25630	I35_RS25630	I35_5843	I35_2975	BCAM2008	-2.4800	1.1074E-06	tetratricopeptide repeat protein
I35_RS25635	I35_RS25635	I35_5844	I35_2974	BCAM2009	-2.0305	2.2585E-03	Fe2+-dependent dioxygenase
I35_RS25785	I35_RS25785	I35_5874	I35_2942	BCAM2039	4.1620	7.2872E-09	winged helix-turn-helix domain-containing protein
I35_RS25820	*asnB*	I35_5880	I35_2505	BCAM2044	4.8229	1.1410E-14	asparagine synthase (glutamine-hydrolyzing)
I35_RS25975	*lysA*	I35_5912	I35_7169	BCAM2076	-1.7632	1.3819E-05	diaminopimelate decarboxylase
I35_RS26310	I35_RS26310				2.0032	1.1978E-03	helix-turn-helix transcriptional regulator
I35_RS36125	I35_RS36125				1.2937	6.0422E-05	helix-turn-helix transcriptional regulator
I35_RS26550	I35_RS26550	I35_6022	I35_7051	BCAM2140	3.2664	2.2374E-12	HlyD family efflux transporter periplasmic adaptor subunit
I35_RS26555	I35_RS26555	I35_6023	I35_7050	BCAM2141	2.9741	2.9311E-23	type I secretion system permease/ATPase
I35_RS26560	I35_RS26560	I35_6024	I35_7049	BCAM2142	3.7213	3.8095E-23	TolC family protein
I35_RS26565	*adhA*	I35_6025	I35_7048	BCAM2143	3.4421	2.4156E-53	cable pilus-associated adhesin AdhA
I35_RS26615	I35_RS26615	I35_6035	I35_7037	BCAM2153	2.2850	2.0677E-14	cytochrome P450
I35_RS26620	I35_RS26620	I35_6036	I35_7036	BCAM2154	2.0264	9.8120E-09	YodC family protein
I35_RS26695	I35_RS26695	I35_6051	I35_7020	BCAM2169	3.1968	9.5901E-24	autotransporter-associated beta strand repeat-containing protein
I35_RS26885	I35_RS26885	I35_6093	I35_6978	BCAM2207	1.0926	7.8379E-03	hypothetical protein
I35_RS27110	I35_RS27110			BCAM2251A	1.4175	2.6616E-11	hypothetical protein
I35_RS27295	I35_RS27295	I35_6180	I35_6889		1.6995	4.9529E-11	DUF4148 domain-containing protein
I35_RS27385	I35_RS27385	I35_6198	I35_6870	BCAM2307	4.3636	3.7458E-13	M36 family metallopeptidase
I35_RS27390	I35_RS27390	I35_6199	I35_6869	BCAM2308	3.1446	1.1254E-04	M20/M25/M40 family metallo-hydrolase
I35_RS27520	I35_RS27520	I35_6227	I35_6839	BCAM2334	5.5514	1.6712E-05	efflux RND transporter periplasmic adaptor subunit
I35_RS27525	I35_RS27525	I35_6228	I35_6838	BCAM2335	4.0191	9.1679E-11	efflux transporter outer membrane subunit
I35_RS27530	I35_RS27530	I35_6229	I35_6837	BCAM2336	3.3011	6.3639E-06	glycosyltransferase family 2 protein
I35_RS27535	I35_RS27535	I35_6230	I35_6836	BCAM2337	3.2112	3.7196E-07	DHA2 family efflux MFS transporter permease subunit
I35_RS27540	I35_RS27540	I35_6231	I35_6835	BCAM2338	3.3094	1.3085E-05	nucleotide disphospho-sugar-binding domain-containing protein
I35_RS27545	I35_RS27545	I35_6232	I35_6834	BCAM2339	3.4068	1.2825E-06	isoprenylcysteine carboxylmethyltransferase family protein
I35_RS27550	I35_RS27550	I35_6233	I35_6833	BCAM2340	4.1674	4.2525E-36	alpha/beta hydrolase
I35_RS27725	I35_RS27725	I35_6270	I35_4109	BCAM2374	-2.3661	9.3361E-03	methyl-accepting chemotaxis protein
I35_RS27990	I35_RS27990	I35_6318	I35_1481	BCAM2419	3.1658	5.4837E-26	OmpA family protein
I35_RS27995	I35_RS27995	I35_6319	I35_1479	BCAM2420	1.1129	2.3964E-07	DUF2827 domain-containing protein
I35_RS28000	I35_RS28000	I35_6320	I35_1478	BCAM2421	1.2284	6.0701E-07	DUF2827 domain-containing protein
I35_RS28015	I35_RS28015	I35_6323	I35_1475	BCAM2424	1.3125	1.8834E-05	FkbM family methyltransferase
I35_RS28020	I35_RS28020	I35_6324	I35_1474	BCAM2425	4.3830	3.6767E-09	hypothetical protein
I35_RS28025	I35_RS28025	I35_6325	I35_1473	BCAM2426	3.6041	5.2658E-31	EAL domain-containing protein
I35_RS28120	I35_RS28120	I35_6344	I35_1451	BCAM2444	3.5382	5.2467E-71	SMP-30/gluconolaconase/LRE-like region family protein
I35_RS37495	I35_RS37495				3.5808	1.3349E-25	hypothetical protein
I35_RS37095	I35_RS37095				1.5183	1.5894E-11	lipid II-degrading bacteriocin
I35_RS29035	I35_RS29035	I35_6522	I35_0003	BCAM2626	1.3703	2.6870E-04	TonB-dependent hemoglobin/transferrin/lactoferrin family receptor
I35_RS29590	I35_RS29590	I35_6632	I35_0221	BCAM2746	-1.0554	9.8814E-05	carbon starvation CstA family protein
I35_RS30000	I35_RS30000	I35_6716	I35_3062	BCAM2836	-1.7947	9.7803E-06	diguanylate cyclase
I35_RS30005	I35_RS30005	I35_6717	I35_3061		-2.5705	1.0186E-09	chemotaxis protein CheC
I35_RS30010	I35_RS30010				-1.9930	5.9397E-08	response regulator
**pC3**							
I35_RS30660	I35_RS30660	I35_7127	I35_5826	BCAS0130	-1.2098	3.5846E-03	glycine betaine ABC transporter substrate-binding protein
I35_RS30765	I35_RS30765	I35_7149	I35_5849	BCAS0151	-1.4729	8.3129E-06	hypothetical protein
I35_RS30770	I35_RS30770	I35_7150	I35_5850	BCAS0152	-1.1640	2.2585E-03	alpha/beta hydrolase
I35_RS31110	I35_RS31110	I35_7216	I35_5921	BCAS0204	5.3704	6.9146E-03	ATP-binding cassette domain-containing protein
I35_RS31115	I35_RS31115	I35_7217	I35_5922	BCAS0205	4.4797	6.3404E-03	TauD/TfdA family dioxygenase
I35_RS31120	I35_RS31120	I35_7218	I35_5923	BCAS0206	5.2038	2.3282E-04	class I SAM-dependent methyltransferase
I35_RS31125	I35_RS31125	I35_7219	I35_5924	BCAS0207	3.5842	8.1172E-04	citrate/2-methylcitrate synthase

I35_RS31130	I35_RS31130	I35_7220	I35_5925	BCAS0208	4.8193	4.0398E-08	acyl-CoA dehydrogenase
I35_RS31135	I35_RS31135	I35_7221	I35_5926	BCAS0209	4.1559	2.5251E-03	hypothetical protein
I35_RS31140	I35_RS31140	I35_7222	I35_5927	BCAS0210	5.1039	4.0663E-06	acyl-CoA ligase (AMP-forming), exosortase A system-associated
I35_RS31145	I35_RS31145	I35_7223	I35_5928	BCAS0211	3.5760	2.2513E-04	PLP-dependent decarboxylase
I35_RS31150	I35_RS31150	I35_7224	I35_5929	BCAS0212	4.4001	1.7833E-04	3-oxoacyl-ACP synthase
I35_RS31155	I35_RS31155	I35_7225	I35_5930	BCAS0213	4.3706	2.6683E-08	ferritin-like domain-containing protein
I35_RS31160	I35_RS31160	I35_7226	I35_5931	BCAS0214	6.8868	7.4184E-06	hypothetical protein
I35_RS31165	I35_RS31165	I35_7227	I35_5932	BCAS0215	5.9216	5.1415E-04	hypothetical protein
I35_RS31170	I35_RS31170	I35_7228	I35_5933	BCAS0216	6.8110	5.5229E-06	acyl carrier protein
I35_RS31175	I35_RS31175	I35_7229	I35_5934	BCAS0217	4.0175	6.5857E-07	hypothetical protein
I35_RS31180	I35_RS31180	I35_7230	I35_5935	BCAS0218	7.5892	3.8090E-04	hypothetical protein
I35_RS31185	I35_RS31185	I35_7231	I35_5936	BCAS0219	7.3532	4.3337E-07	outer membrane lipoprotein-sorting protein
I35_RS31190	I35_RS31190	I35_7232	I35_5937	BCAS0220	5.1711	5.6356E-06	ABC transporter permease
I35_RS31195	I35_RS31195	I35_7233	I35_5938		5.7592	1.7961E-03	ABC transporter ATP-binding protein
I35_RS31200	I35_RS31200	I35_7234	I35_5939	BCAS0222	7.3717	1.0186E-09	fatty acyl-AMP ligase
I35_RS31205	I35_RS31205	I35_7236	I35_5941	BCAS0223	4.9704	4.4985E-04	fatty acid desaturase
I35_RS31210	I35_RS31210	I35_7237	I35_5942		5.2708	2.0181E-07	diiron oxygenase
I35_RS31215	I35_RS31215	I35_7238	I35_5943	BCAS0225	3.4380	9.3540E-28	LysR substrate-binding domain-containing protein
I35_RS31545	I35_RS31545	I35_7308	I35_6019	BCAS0292	5.7951	2.3022E-49	AidA/PixA family protein
I35_RS31550	I35_RS31550	I35_7309	I35_6020	BCAS0293	5.4584	5.7528E-93	inclusion body family protein
I35_RS31680	I35_RS31680	I35_7336	I35_6048	BCAS0319	3.0035	1.3441E-03	xanthine dehydrogenase family protein molybdopterin-binding subunit
I35_RS31690	I35_RS31690	I35_7338	I35_6050	BCAS0321	3.6126	9.2972E-15	autotransporter-associated beta strand repeat-containing protein
I35_RS31750	I35_RS31750	I35_7352	I35_6064	BCAS0334	2.7499	9.9507E-23	response regulator
I35_RS31755	I35_RS31755	I35_7354	I35_6066	BCAS0335	6.8535	1.5910E-60	YadA-like family protein
I35_RS31760	I35_RS31760	I35_7355	I35_6067	BCAS0336	2.4665	7.2079E-08	VOC family protein
I35_RS32220	I35_RS32220	I35_7447	I35_6162	BCAS0409	4.3800	2.0958E-65	M4 family metallopeptidase
I35_RS32225	I35_RS32225	I35_7448	I35_6163		1.8484	7.7376E-09	hypothetical protein
I35_RS32230	I35_RS32230	I35_7449	I35_6164		1.3895	3.0560E-03	hypothetical protein
I35_RS32235	I35_RS32235				1.7646	3.8990E-14	glycosyltransferase family 9 protein
I35_RS32240	I35_RS32240	I35_7450	I35_6165		2.1082	3.1133E-28	tetratricopeptide repeat-containing glycosyltransferase family protein
I35_RS32245	I35_RS32245	I35_7451	I35_6166		1.2914	1.6459E-10	FkbM family methyltransferase
I35_RS32275	I35_RS32275	I35_7454	I35_6169	BCAS0412	1.4322	4.7684E-05	hypothetical protein
I35_RS32815	I35_RS32815	I35_7563	I35_6290		1.2944	4.0831E-03	hypothetical protein
I35_RS32825	I35_RS32825	I35_7564	I35_6291		1.5153	1.0096E-08	hypothetical protein
I35_RS33945	I35_RS33945	I35_7792	I35_6531	BCAS0628	3.3998	6.8121E-32	TetR/AcrR family transcriptional regulator
I35_RS33950	I35_RS33950	I35_7793	I35_6532	BCAS0629	2.9229	2.1524E-24	hypothetical protein
I35_RS33955	I35_RS33955	I35_7794	I35_6533	BCAS0630	2.9021	2.5885E-06	Nramp family divalent metal transporter
I35_RS34160	I35_RS34160	I35_7835	I35_6576		1.7841	5.2408E-03	gallate dioxygenase
I35_RS38030	I35_RS38030				3.4321	5.0446E-41	DUF1629 domain-containing protein
I35_RS38035	I35_RS38035				2.0507	6.2762E-19	RHS repeat domain-containing protein

[a] According to the *Burkholderia* Genome Database (https://burkholderia.com). Adjacent genes are denoted by the same color

[b] $\log_2$ fold change of the transcript levels in the *hns*$_{1.2}$ deletion mutant relative to parental cells
