# Supplementary material for: A functional interplay of hns genes governs the control of the motile-to-biofilm lifestyle switch in Burkholderia cenocepacia
Source: Microbiol Spectr. 2026 Sep 2;14(10):e00344-26. doi: 10.1128/spectrum.00344-26 (PMC13689386; doi:10.1128/spectrum.00344-26)
Supplement: Supplementary file 2 [file spectrum.00344-26-s0002.pdf]

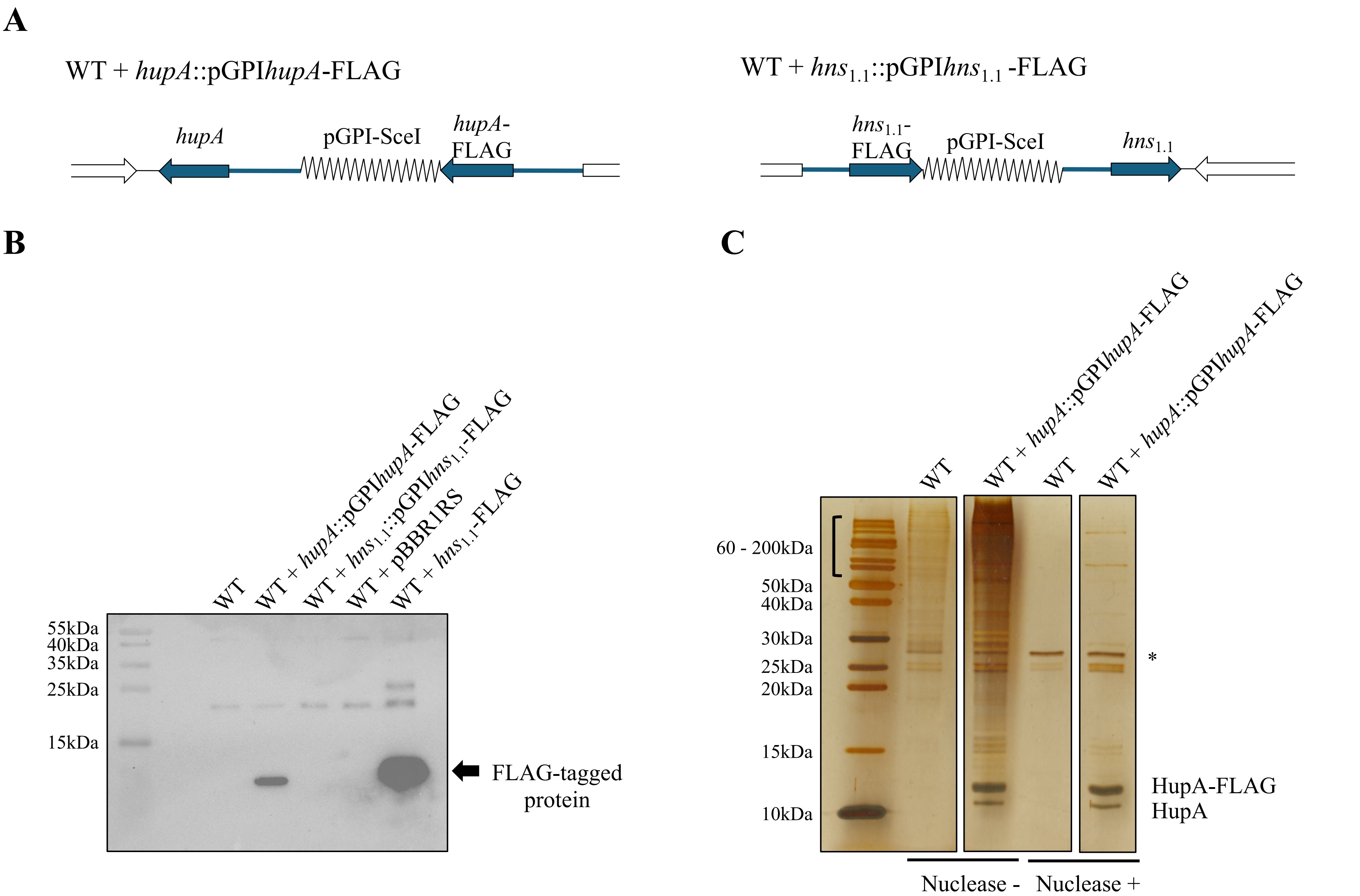

Figure S1. Expression and purification of FLAG-tagged proteins in *B. cenocepacia* H111. A) Schematic representation of chromosome 1 around the *hupA* and $hns_{1.1}$ genes expected to be found after the integration of the pGPI-SceI derivatives. The homologous regions are represented by blue arrows (coding sequence) and lines (upstream region). **B)** Saturated cultures were diluted in fresh LLB medium and grown to $OD_{600nm} \simeq 0.5$. Cells were harvested, washed and lysed by sonication, and the cleared lysates were used in co-IP experiments with an anti-FLAG affinity gel. The volumes of both affinity gel and elution buffer were proportional to the total protein concentration in the cleared lysates, as determined by Bradford reagent. 10 µL each eluate were resolved by SDS-PAGE, the proteins were transferred to a membrane, and the blot was incubated with anti-FLAG and anti-mouse conjugated with peroxidase. The image was obtained by merging the chemiluminescent signal generated from peroxidase with the colorimetric signal of a prestained protein ladder. A band observed in all samples may correspond to the α-FLAG light chain. **C)** Two elution samples described in B were treated or not with 10U/µL Universal Nuclease (ThermoFischer Scientific) for 30 min at room temperature and resolved by SDS-PAGE. The gel was silver stained. * indicates a protein band at a position expected for the nuclease added to the elution samples.

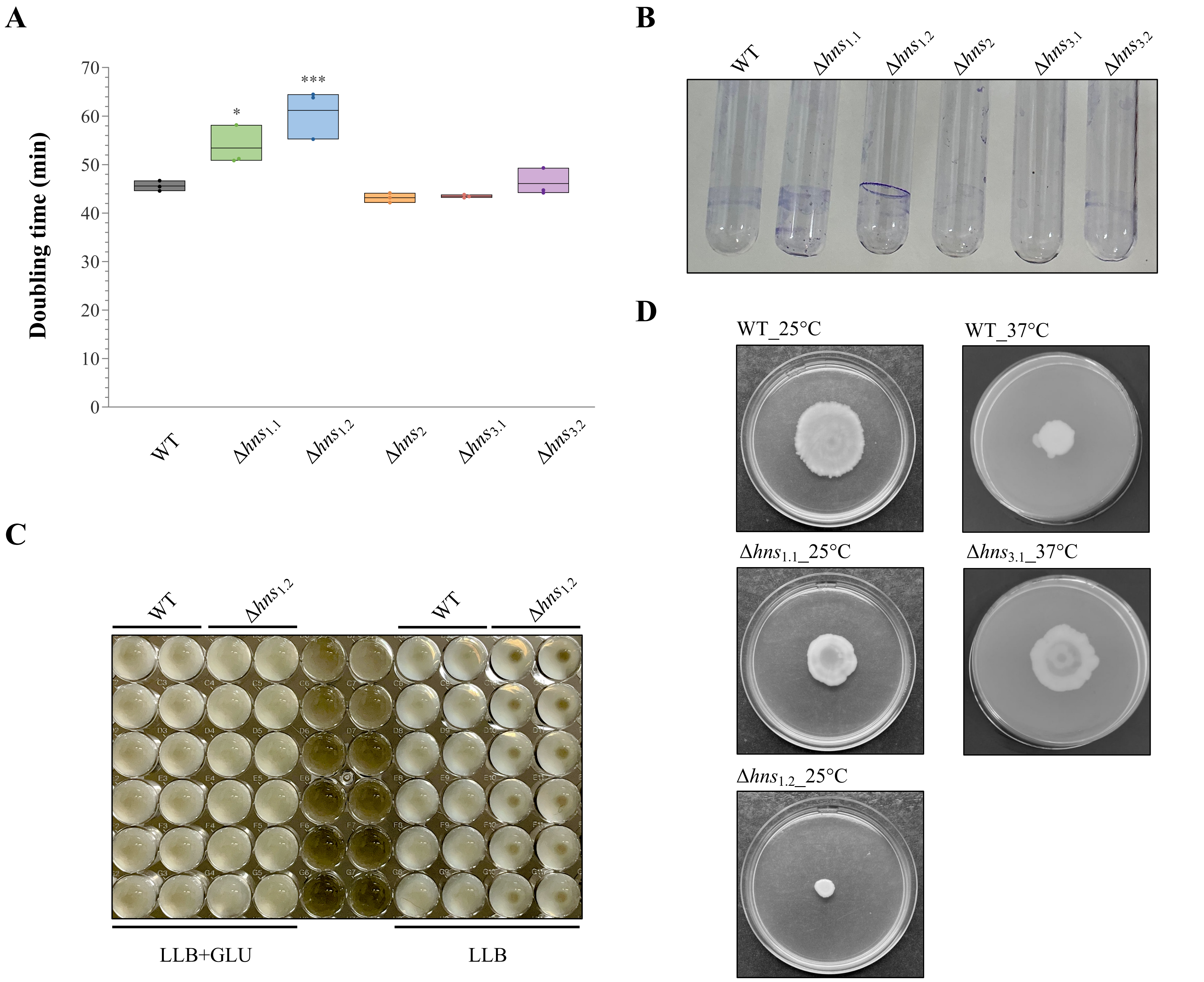

Figure S2. Grown, biofilm formation and motility in the *hns* mutants and parental cells. A) Doubling time of each strain calculated using the growth curves shown in Fig 2A. The initial four hours of the growth curves were considered to estimate the doubling time, as cultures were at the exponential growth phase. Data are mean ± standard deviation of three independent experiments. Statistical analysis was carried out using one-way ANOVA followed by Dunnett's test to compare the mutant strains to a single control (WT) (*, $p < 0.05$; ***, $p < 0.001$). **B)** Strains were grown for 16 h at 37°C in LLB medium under shaking condition (200 rpm), and attached cells were washed, stained with 1% crystal violet and imaged. **C**) Representative image of the bottom of a microtiter polystyrene dish containing cultures grown in LLB medium supplemented or not with glucose (GLU) under static conditions for 24 h at 37°C. **D**) Representative images of the swarming motility after 72 h at either 25 or 37°C. Only mutant strains displaying significant difference in motility with respect to parental cells are shown.
