# Supplementary material for: A functional interplay of hns genes governs the control of the motile-to-biofilm lifestyle switch in Burkholderia cenocepacia
Source: Microbiol Spectr. 2026 Sep 2;14(10):e00344-26. doi: 10.1128/spectrum.00344-26 (PMC13689386; doi:10.1128/spectrum.00344-26)
Supplement: Supplementary file 3 [file spectrum.00344-26-s0003.pdf]

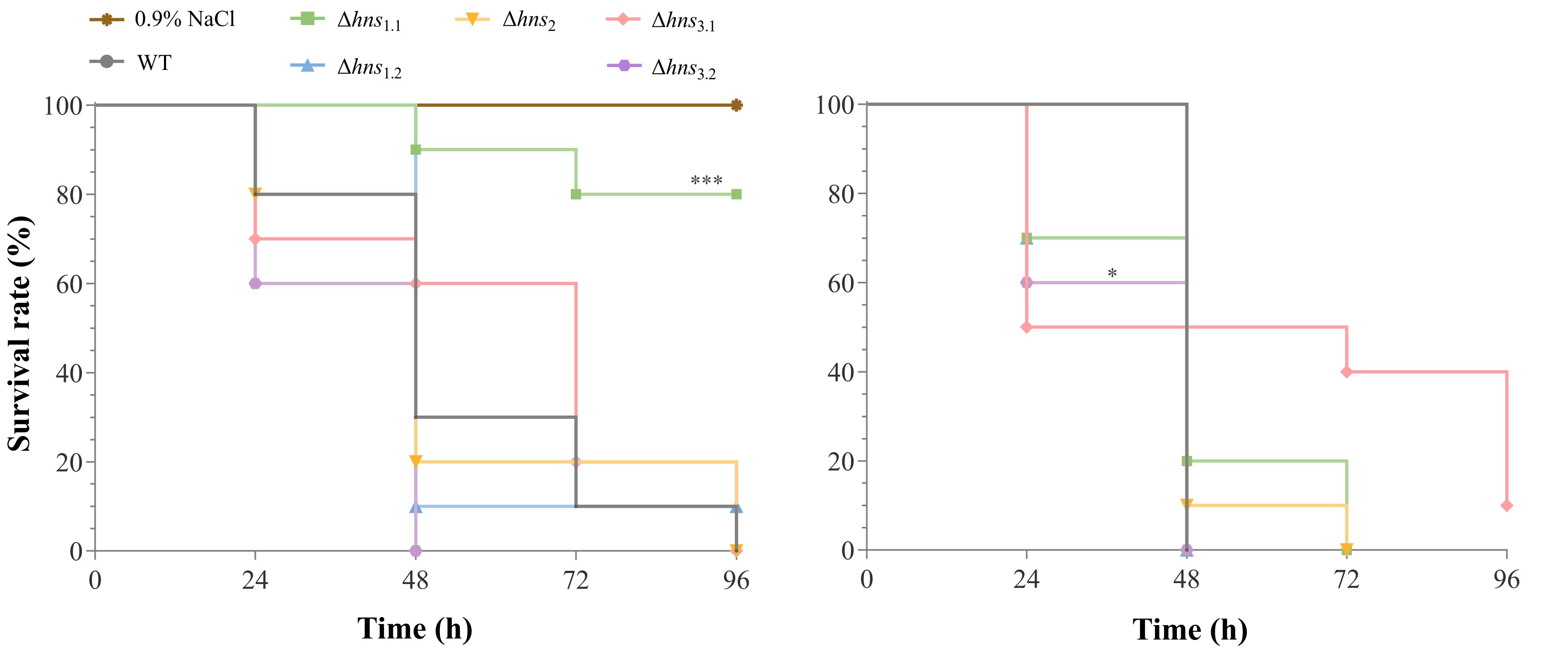

Figure S3. Additional *G. mellonella* killing experiments. *G. mellonella* at the larval stage was infected with the *hns* mutants and parental cells grown to exponential phase ($OD_{600nm} \simeq 0.5$) and diluted to approximately 10^7 CFU/ mL. The 10 µL aliquots injected in each larva was estimated as containing: 6.6×10^5 (WT), 3.9×10^5 (Δ*hns*$_{1.1}$), 3.8×10^5 (Δ*hns*$_{1.2}$), 4.7×10^5 (Δ*hns*$_2$), 4.6×10^5 (Δ*hns*$_{3.1}$) and 4.1×10^6 (Δ*hns*$_{3.2}$) for the experiment shown at the left panel, and 8.2×10^5 (WT), 8.4×10^5 (Δ*hns*$_{1.1}$), 8.2×10^5 (Δ*hns*$_{1.2}$), 6.4×10^5 (Δ*hns*$_2$), 7.4×10^5 (Δ*hns*$_{3.1}$) and 7.5×10^5 (Δ*hns*$_{3.2}$) for the experiment shown at the right panel. The Kaplan-Meier survival curves were analyzed using a Log-rank (Mantel-Cox) test (*, $p < 0.05$; ***, $p < 0.001$).

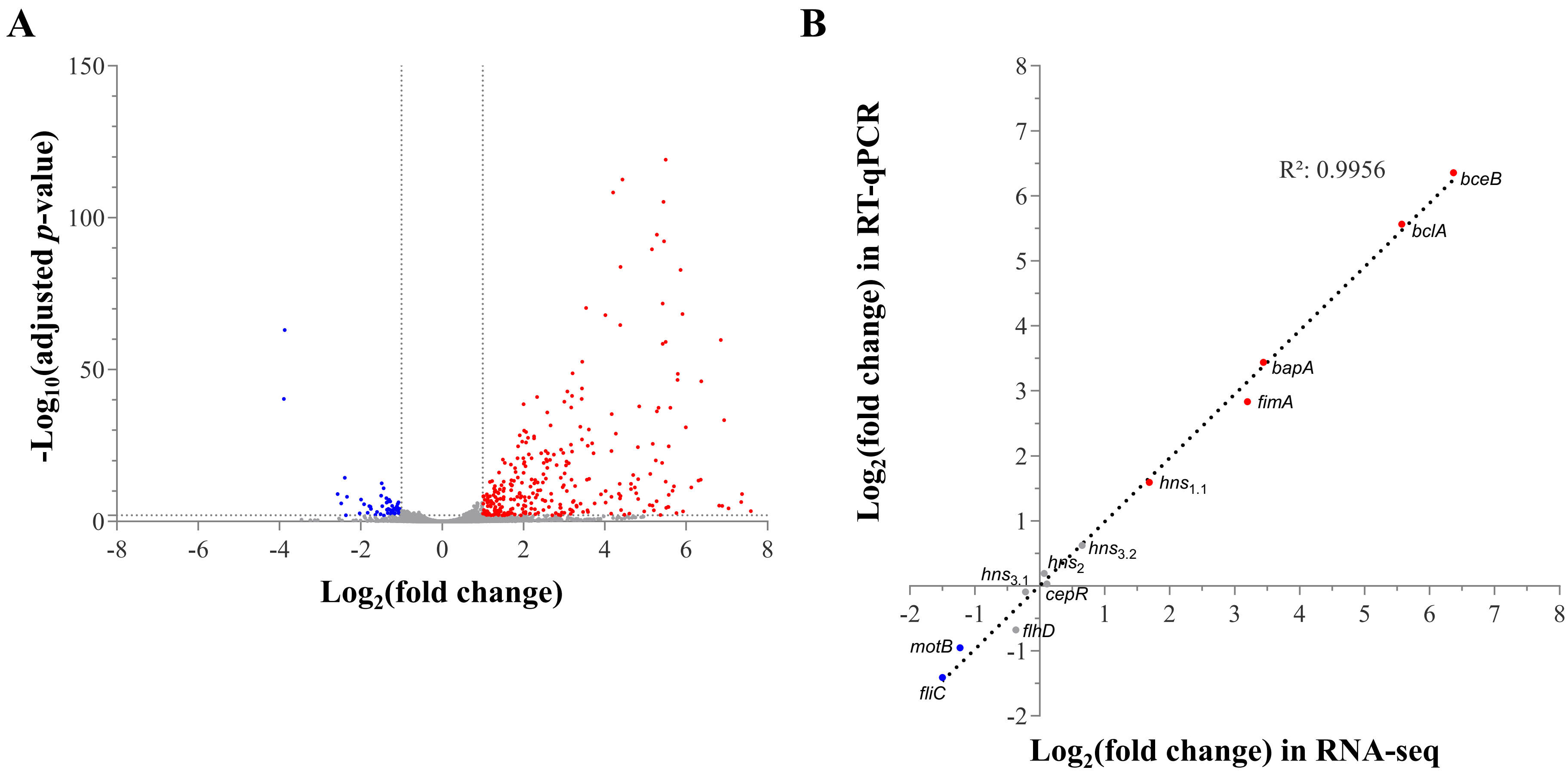

Figure S4. Gene expression analyses comparing the *hns*$_{1.2}$ deletion mutant with parental cells. A) Volcano plot with the RNA-seq data comparing the transcriptome of cells deleted for *hns*$_{1.2}$ with that of parental cells. Upregulated and downregulated genes are denoted in red and blue, respectively, while genes not considered differentially expressed are shown in gray. Doted lines indicate the cutoffs used for considering genes as differentially expressed between the two strains ($\log_2$ fold change $\leq$ -1 or $\log_2$ fold change $\geq$ 1 and adjusted *p*-value $\leq$ 0.01). **B**) Correlation between RNA-seq and RT-qPCR data with respect to the expression of selected genes. For the RT-qPCR experiments, we analyzed genes related to flagellum (*fliC* and *motB*), fimbria (*fimA*), cepacian exopolysaccharide (*bceB*), adhesins (*bclA* and *bapA*) and the regulatory cascade for gene expression control (*hns*$_{1.1}$), which were considered differentially expressed between the Δ*hns*$_{1.2}$ mutant and parental cells. In addition, *flhD*, *cepR* and the *hns* genes located on chromosome 2 and the megaplasmid pC3 were included as genes whose expression levels were not impacted by *hns*$_{1.2}$ deletion.

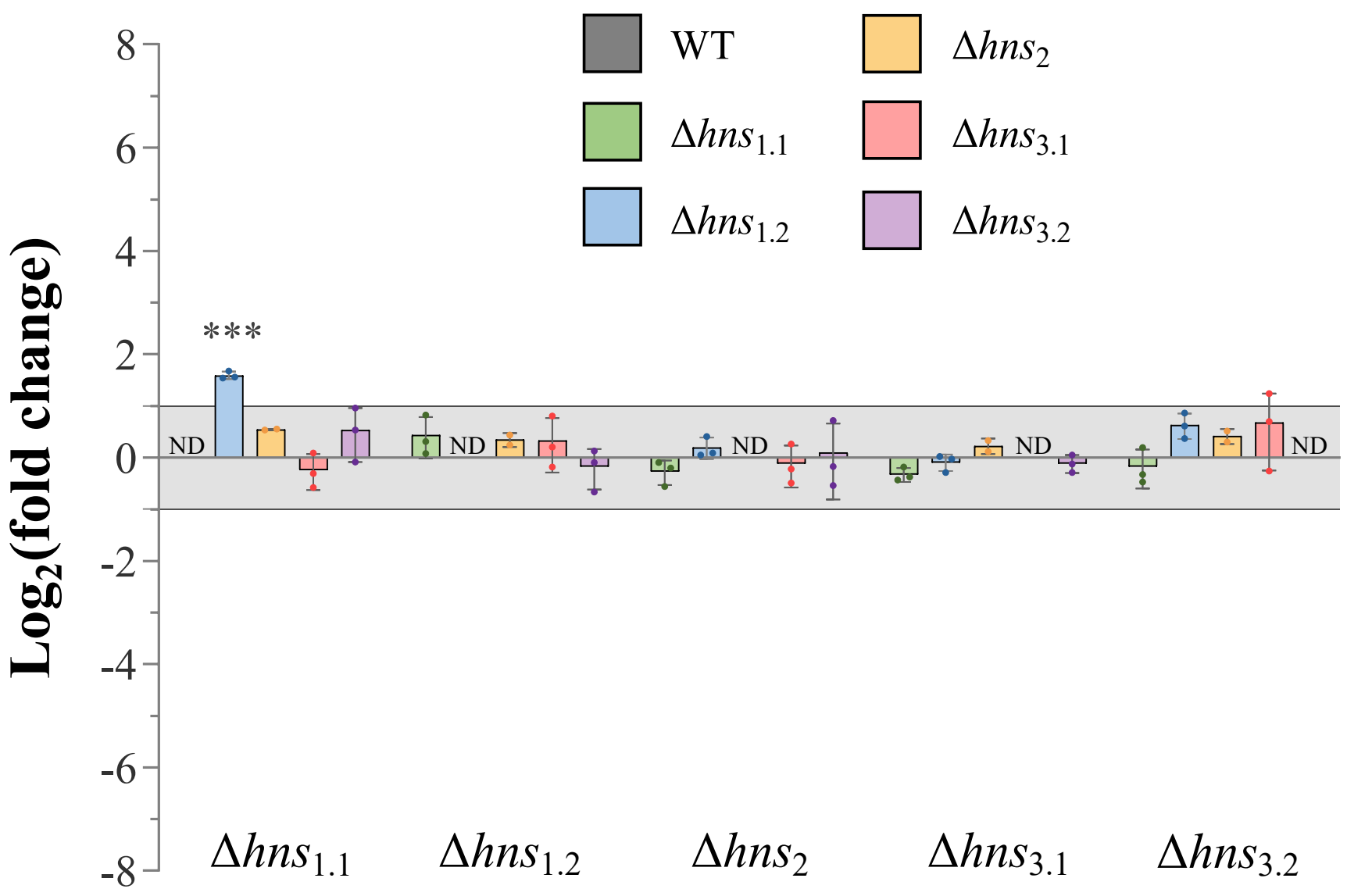

Figure S5. Effect of deleting an *hns* gene on the transcript levels of other *hns* genes. Saturated cultures were diluted in fresh LLB medium and grown to exponential phase ($OD_{600nm} \simeq 0.5$). RT-qPCR experiments were performed starting with total RNA samples, and results were normalized using the *rpoD* gene as an endogenous control and presented as the log_2 fold change of the transcript levels in an *hns* deletion mutant relative to that of parental cells. Data are mean ± standard deviation of three independent experiments. Statistical analysis was carried out using one-way ANOVA followed by Dunnett's test to compare the mutant strains to a single control (WT) (***, $p < 0.001$). Transcript of an *hns* gene was not detected when the corresponding gene was deleted and is denoted as ND.

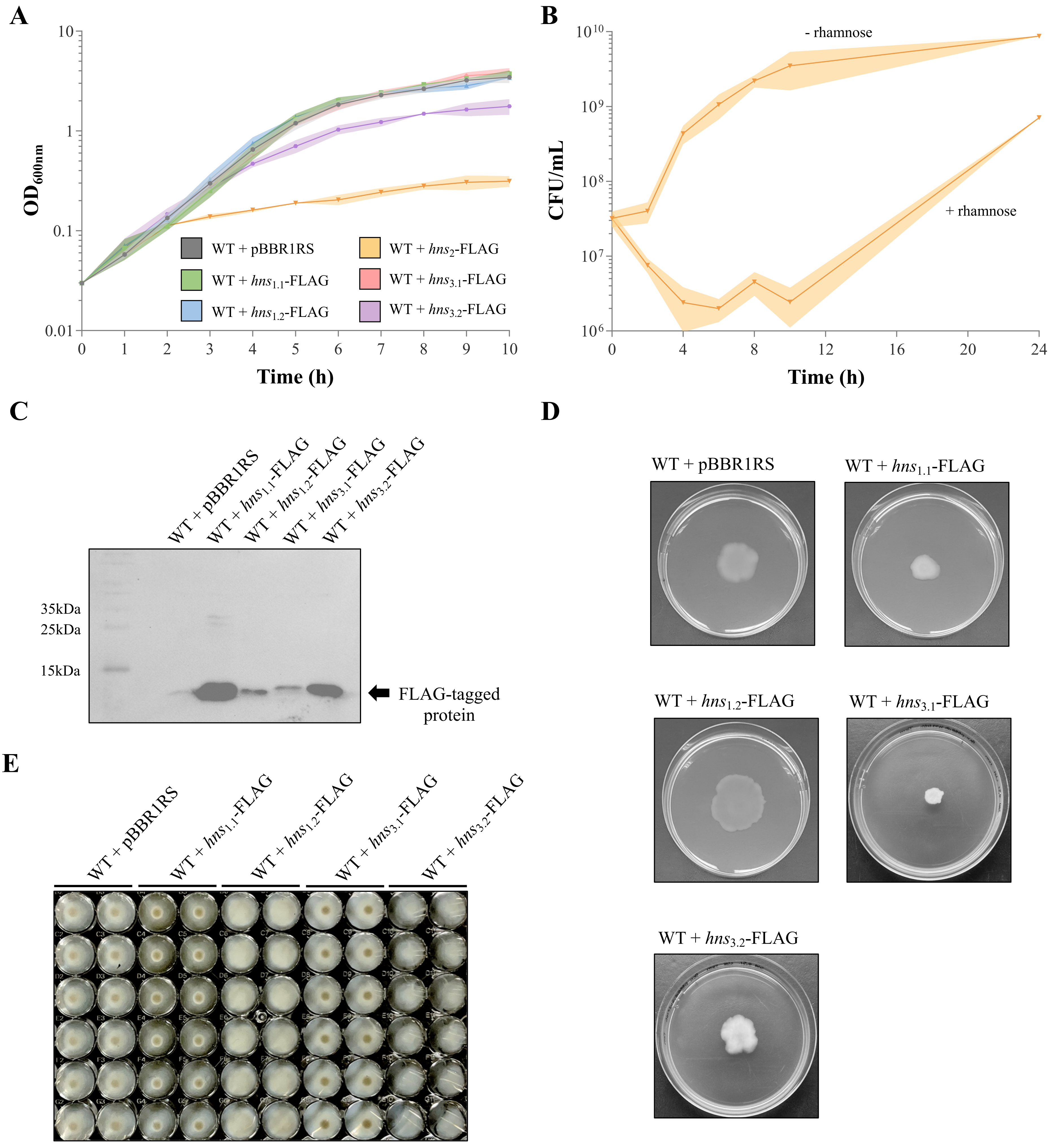

Figure S6. Production of FLAG-tagged H-NS proteins and the effect on bacterial physiology. A) Growth curves of strains producing FLAG-tagged H-NS proteins. Saturated cultures were diluted in fresh LLB medium supplemented with 0.5% rhamnose, and OD_{600nm} was measured at the indicated time points. Data are mean values ± standard deviation of three independent experiments. Doubling time was not calculated because the initial points in the growth curve of cells harboring *hns*$_2$-FLAG could not be fitted with an exponential trendline. **B)** Viability of cells constructed to produce H-NS$_2$-FLAG. Saturated cultures were diluted in fresh LLB medium supplemented or not with 0.5% rhamnose. At the indicated time points, the cultures were serially diluted and plated for CFU counting. Data are mean values ± standard deviation of three independent experiments. **C)** H-NS-FLAG levels determined by western blotting. Saturated cultures were diluted in fresh LLB medium supplemented with 0.5% rhamnose and grown to $OD_{600nm} \simeq 0.5$. Cells were harvested, washed and lysed by sonication. Cleared lysates (50 µg) were resolved by SDS-PAGE, the proteins were transferred to a membrane, and the blot was incubated with anti-FLAG and anti-mouse conjugated with peroxidase. The image was obtained by merging the chemiluminescent signal generated from the peroxidase with the colorimetric signal of the prestained protein ladder. **D)** Representative images of the swarming motility after 72 h at 25°C. **E)** Representative image of the bottom of a microtiter polystyrene dish containing cultures grown under static conditions for 24 h at 37°C.

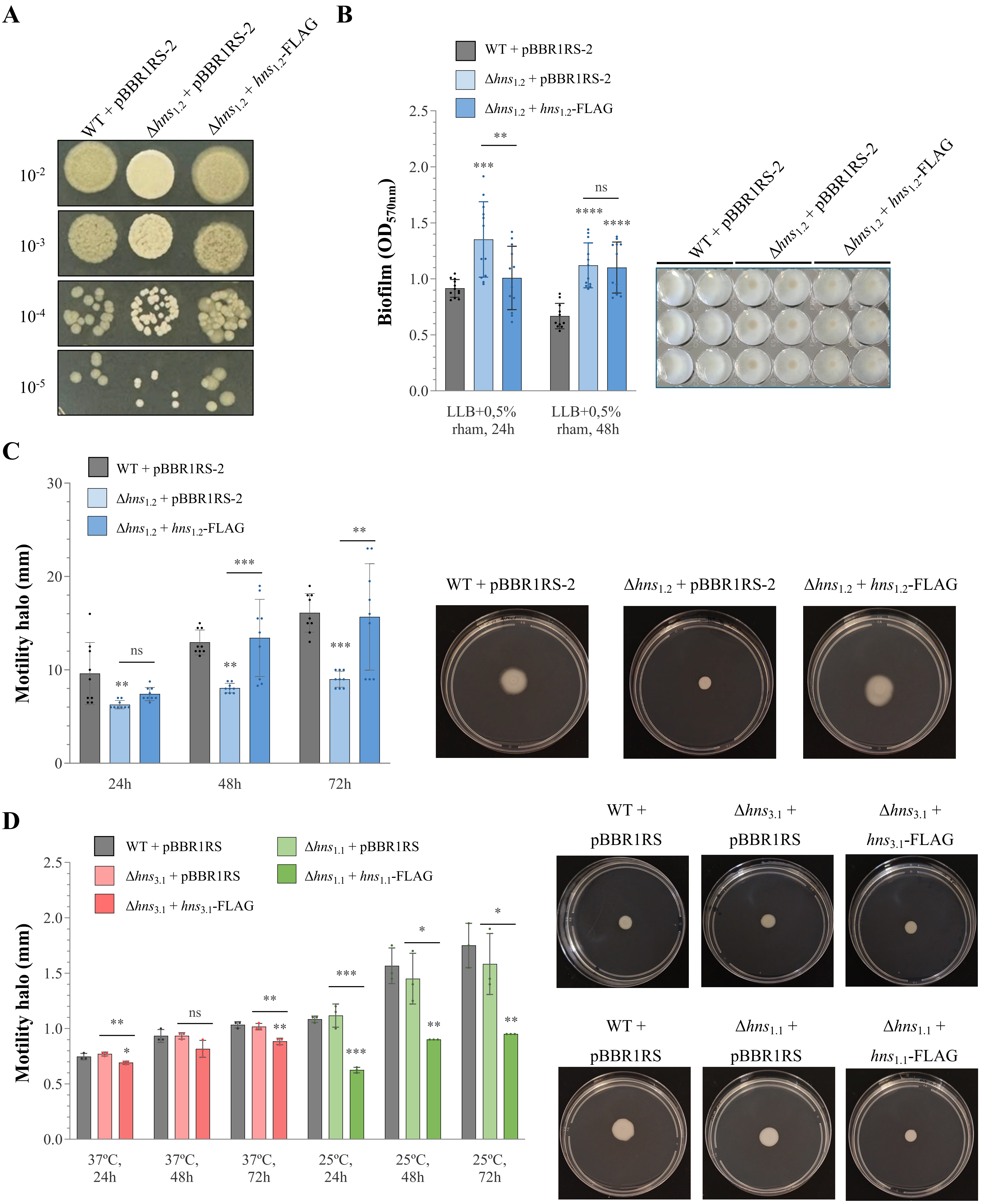

Figure S7. Effect of a plasmid-encoded *hns* gene on the physiology of *hns* mutant strains. A) Colony morphology. Saturated cultures were diluted in fresh LLB medium supplemented with 0.5% rhamnose, grown to exponential phase ($OD_{600nm} \simeq 0.5$), serially diluted and spotted on a rhamnose-containing LLB agar plate. The plate was imaged after 72 h at 25°C. **B)** Biofilm formation. Saturated cultures were diluted in fresh LLB medium supplemented with 0.5% rhamnose and grown in microtiter polystyrene dishes under static conditions. At the indicated time points, attached cells were stained with 1% crystal violet, and OD_{570nm} was determined. Data are mean values ± standard deviation of three independent experiments, with four technical replicates each. **C - D)** Swarming motility. Saturated cultures were diluted in fresh LLB medium supplemented with 0.5% rhamnose, grown to $OD_{600nm} = 0.4$, and spotted on rhamnose-containing semisolid plates. Diameter of motility halo was determined at the indicated time points, and data are mean values ± standard deviation. Statistical analysis was carried out using one-way ANOVA followed by Tukey's test for multiple comparisons. Asterisk indicated above or below a bar indicates significant difference with respect to parental cells harboring an empty vector. For the comparison of the deletion mutant carrying the plasmid-encoded *hns* gene with the same mutant strain harboring the empty vector, either asterisk or ns (no significant change) is indicated above or below a line. (*, $p < 0.05$; **, $p < 0.01$; ***, $p < 0.001$; ****, $p < 0.0001$). Representative images of the swarming motility after 72 h at either 25 or 37°C are shown.
