# Supplementary material for: A functional interplay of hns genes governs the control of the motile-to-biofilm lifestyle switch in Burkholderia cenocepacia
Source: Microbiol Spectr. 2026 Sep 2;14(10):e00344-26. doi: 10.1128/spectrum.00344-26 (PMC13689386; doi:10.1128/spectrum.00344-26)
Supplement: Supplementary file 4 [file spectrum.00344-26-s0004.pdf]

Table S3. List of oligonucleotides designed and used in this study

Primer name	Sequence (5' – 3')	Usage
hupA-FLAG_F1	TTA T**GG TAC C**TT AAC AAG GGA TTG CGG GAG AC	Amplification of the *hupA* coding sequence and its 358 bp upstream region. A **KpnI restriction site** was added to clone the amplicon into the pGPI-SceI plasmid
hupA-FLAG_R1	TTA CTT GTC GTC GTC GTC CTT GTA GTC ACG CTT GTT GAC GGC GTC	Amplification of the *hupA* coding sequence and its 358 bp upstream region. The FLAG nucleotide sequence was incorporated to the primer, and the XbaI restriction site from the pJET1.2 vector was used to clone the fragment into the pGPI-SceI plasmid
hns$_{1.1}$-FLAG_F1	**GGT ACC** ACG CAA CGG ATT TAT TTC	Amplification of the *hns*$_{1.1}$ coding sequence and its 261 bp upstream region. A **KpnI restriction site** was added to clone the amplicon into the pGPI-SceI plasmid
hns$_{1.1}$-FLAG_R1	TCA CTT GTC GTC GTC GTC CTT GTA GTC CTC GAT CAG GAA ACG ATC	Amplification of the *hns*$_{1.1}$ coding sequence and its 261 bp upstream region. The FLAG nucleotide sequence was incorporated to the primer, and the XbaI restriction site from the pJET1.2 vector was used to clone the fragment into the pGPI-SceI plasmid
delta*hns*$_{1.1}$_F1	TTA T**GG TAC C**TG TGG CCG ATT GCG AAT G	Amplification of a 514 bp upstream fragment for deleting *hns*$_{1.1}$. A **KpnI restriction site** was added to clone the amplicon into the pGPI-SceI plasmid
delta*hns*$_{1.1}$_R1	CAG GAA ACG GAG TTT CGC GTA TTG AGA C	Amplification of a 514 bp upstream fragment for deleting *hns*$_{1.1}$. This sequence partially overlaps with the delta*hns*$_{1.1}$_F2 primer
delta*hns*$_{1.1}$_F2	GCG AAA CTC CGT TTC CTG ATC GAG TGA C	Amplification of a 647 bp downstream fragment for deleting *hns*$_{1.1}$. This sequence partially overlaps with the delta*hns*$_{1.1}$_R1 primer

delta*hns*$_{1.1}$_R2	CGA AAA GCG TGT CGA CAC	Amplification of a 647 bp downstream fragment for deleting *hns*$_{1.1}$. An internal **EcoRI restriction site** was used to clone the amplicon into the pGPI-SceI plasmid
delta*hns*$_{1.2}$_F1	TTA T**GG TAC C**CG GCG ATT TCG CGT TCT G	Amplification of a 715 bp upstream fragment for deleting *hns*$_{1.2}$. A **KpnI restriction site** was added to clone the amplicon into the pGPI-SceI plasmid
delta*hns*$_{1.2}$_R1	TTC GCG GTT GGC CAG CAG GTC CTT GTA AG	Amplification of a 715 bp upstream fragment for deleting *hns*$_{1.2}$. This sequence partially overlaps with the delta*hns*$_{1.2}$_F2 primer
delta*hns*$_{1.2}$_F2	CTG CTG GCC AAC CGC GAA CAG TTT GCG	Amplification of a 695 bp downstream fragment for deleting *hns*$_{1.2}$. This sequence partially overlaps with the delta*hns*$_{1.2}$_R1 primer
delta*hns*$_{1.2}$_R2	TTA T**GA ATT C**CG TCA GCG CAA CGA TCA G	Amplification of a 695 bp downstream fragment for deleting *hns*$_{1.2}$. A **EcoRI restriction site** was added to clone the amplicon into the pGPI-SceI plasmid
delta*hns*$_2$_F1	TTA T**TC TAG A**AT TCG CAT CAC CGA CGA TC	Amplification of a 543 bp upstream fragment for deleting *hns*$_2$. A **XbaI restriction site** was added to clone the amplicon into the pGPI-SceI plasmid
delta*hns*$_2$_R1	GAA ACG TCC GTA GGT CGC CAT ATG GAT G	Amplification of a 543 bp upstream fragment for deleting *hns*$_2$. This sequence partially overlaps with the delta*hns*$_2$_F2 primer
delta*hns*$_2$_F2	GCG ACC TAC GGA CGT TTC CTG ATC AAG	Amplification of a 607 bp downstream fragment for deleting *hns*$_2$. This sequence partially overlaps with the delta*hns*$_2$_R1 primer
delta*hns*$_2$_R2	TTA T**GG TAC C**AC GAA AGA CGA CAC GAT C	Amplification of a 607 bp downstream fragment for deleting *hns*$_2$. A **KpnI restriction site** was added to clone the amplicon into the pGPI-SceI plasmid

delta*hns*$_{3.1}$_F1	TTA T**TC TAG A**AA CCG GCA TCC GAA CCT G	Amplification of a 607 bp upstream fragment for deleting *hns*$_{3.1}$. A **XbaI restriction site** was added to clone the amplicon into the pGPI-SceI plasmid
delta*hns*$_{3.1}$_R1	TGA TCG TCC CCT TGT GCT TCG AGT TCC TG	Amplification of a 607 bp upstream fragment for deleting *hns*$_{3.1}$. This sequence partially overlaps with the delta*hns*$_{3.1}$_F2 primer
delta*hns*$_{3.1}$_F2	AGC ACA AGG GGA CGA TCA GGG CTG AAG	Amplification of a 644 bp downstream fragment for deleting *hns*$_{3.1}$. This sequence partially overlaps with the delta*hns*$_{3.1}$_R1 primer
delta*hns*$_{3.1}$_R2	TTA T**GA ATT C**AT CGC GAA CAG CAG GAT C	Amplification of a 644 bp downstream fragment for deleting *hns*$_{3.1}$. A **EcoRI restriction site** was added to clone the amplicon into the pGPI-SceI plasmid
delta*hns*$_{3.2}$_F1	TTA T**TC TAG A**TC GCT TTG CAT CGT GAA G	Amplification of a 599 bp upstream fragment for deleting *hns*$_{3.2}$. A **XbaI restriction site** was added to clone the amplicon into the pGPI-SceI plasmid
delta*hns*$_{3.2}$_R1	AAT CGA TTG CGC TTG TAA GTT GCC ATA TAT CAC	Amplification of a 599 bp upstream fragment for deleting *hns*$_{3.2}$. This sequence partially overlaps with the delta*hns*$_{3.2}$_F2 primer
delta*hns*$_{3.2}$_F2	TTA CAA GCG CAA TCG ATT CCT GAT CCA G	Amplification of a 613 bp downstream fragment for deleting *hns*$_{3.2}$. This sequence partially overlaps with the delta*hns*$_{3.2}$_R1 primer
delta*hns*$_{3.2}$_R2	TTA T**GG TAC C**CA GCG AAC ATT TCC CGT C	Amplification of a 613 bp downstream fragment for deleting *hns*$_{3.2}$. A **KpnI restriction site** was added to clone the amplicon into the pGPI-SceI plasmid
pBBR1_F1	TTA T**AC TAG T**TA AGC GGA TGA ATG GCA G	Amplification of 4008 and 4560 bp fragments from the pBBR1MCS and pBBR1MCS-2 plasmids, respectively. A **SpeI restriction site** was added to fuse the fragment to the rhamnose system amplified from the pSC200 plasmid

pBBR1_R1	TTA T**CT CGA G**GG GTG CCT AAT GAG TGA G	Amplification of 4008 and 4560 bp fragments from the pBBR1MCS and pBBR1MCS-2 plasmids, respectively. A **XhoI restriction site** was added to fuse the fragment to the rhamnose system amplified from the pSC200 plasmid
rhamnose system_F1	TTA T**CT CGA G**GA ACA CTT AAC GGC TGA C	Amplification of 2314 bp fragment from the pSC200 plasmid. A **XhoI restriction site** was added to fuse the fragment to the region amplified from pBBR1MCS and pBBR1MCS-2
rhamnose system_R1	TTA T**AC TAG T**CG TTC GGT CAA GGT TCT G	Amplification of 2314 bp fragment from the pSC200 plasmid. A **SpeI restriction site** was added to fuse the fragment to the region amplified from pBBR1MCS and pBBR1MCS-2
rhamnose system_F2.1	AGA AGA CAT ATG TGA TCC TGC TGA ATT TC	Amplification of a 2177 bp fragment corresponding to the rhamnose system from pSC200. This sequence partially overlaps with the *hns*$_{1.2}$-FLAG_F1 primer
hns$_{1.2}$-FLAG_F1	GGA TCA CAT ATG TCT TCT TAC AAG GAC	Amplification of the *hns*$_{1.2}$ coding sequence. This sequence partially overlaps with the rhamnose system_F2.1 primer
hns$_{1.2}$-FLAG_R1	TTA CTT GTC GTC GTC GTC CTT GTA GTC AAT CGC AAA CTG TTC GCG GTT C	Amplification of the *hns*$_{1.2}$ coding sequence. The FLAG nucleotide sequence was incorporated to the primer
rhamnose system_F2.2	TTG AGA CAT ATG TGA TCC TGC TGA ATT TC	Amplification of a 2177 bp fragment corresponding to the rhamnose system from pSC200. This sequence partially overlaps with the *hns*$_{1.1}$-FLAG F2 primer
hns$_{1.1}$-FLAG_F2	GGA TCA CAT ATG TCT CAA TAC GCG AAA C	Amplification of the *hns*$_{1.1}$ coding sequence. This sequence partially overlaps with the rhamnose system_F2.2 primer
hns$_{1.1}$-FLAG_R2	TCA CTT GTC GTC GTC GTC CTT GTA GTC CTC GAT CAG GAA ACG ATC	Amplification of the *hns*$_{1.1}$ coding sequence. The FLAG nucleotide sequence was incorporated to the primer

rhamnose system_F2.3	GGT CGC CAT ATG TGA TCC TGC TGA ATT TC	Amplification of a 2177 bp fragment corresponding to the rhamnose system from pSC200. This sequence partially overlaps with the *hns*$_{2}$-FLAG F1 primer
hns$_{2}$-FLAG_F1	GGA TCA CAT ATG GCG ACC TAC AGG CAA C	Amplification of the *hns*$_{2}$ coding sequence. This sequence partially overlaps with the rhamnose system_F2.3 primer
hns$_{2}$-FLAG_R1	TCA CTT GTC GTC GTC GTC CTT GTA GTC GTC CTT GAT CAG GAA ACG TC	Amplification of the *hns*$_{2}$ coding sequence. The FLAG nucleotide sequence was incorporated to the primer
rhamnose system_F2.4	CTG GGT CAT ATG TGA TCC TGC TGA ATT TC	Amplification of a 2177 bp fragment corresponding to the rhamnose system from pSC200. This sequence partially overlaps with the *hns*$_{3.1}$-FLAG F1 primer
hns$_{3.1}$-FLAG_F1	GGA TCA CAT ATG ACC CAG GCC CTG CAG	Amplification of the *hns*$_{3.1}$ coding sequence. This sequence partially overlaps with the rhamnose system_F2.4 primer
hns$_{3.1}$-FLAG_R1	TCA CTT GTC GTC GTC GTC CTT GTA GTC GCC CTG ATC GTC CCC GGG	Amplification of the *hns*$_{3.1}$ coding sequence. The FLAG nucleotide sequence was incorporated to the primer
rhamnose system_F2.5	AGT TGC CAT ATG TGA TCC TGC TGA ATT TC	Amplification of a 2177 bp fragment corresponding to the rhamnose system from pSC200. This sequence partially overlaps with the *hns*$_{3.2}$-FLAG F1 primer
hns$_{3.2}$-FLAG_F1	GGA TCA CAT ATG GCA ACT TAC AAG GAA C	Amplification of the *hns*$_{3.2}$ coding sequence. This sequence partially overlaps with the rhamnose system_F2.5 primer
hns$_{3.2}$-FLAG_R1	TCA CTT GTC GTC GTC GTC CTT GTA GTC TTC CTG GAT CAG GAA TCG	Amplification of the *hns*$_{3.2}$ coding sequence. The FLAG nucleotide sequence was incorporated to the primer
FLAG_R1	TTA T**AA GCT T**TC ACT TGT CGT CGT CGT C	Amplification of the rhamnose system with an *hns* coding sequence. A **HindIII restriction site**

		was added to clone the amplicon into pBBR1RS or pBBR1RS-2.
*rpoD*_qPCR_F1	AGG AAG CCG AAG AGG AAG AG	Amplification of *rpoD* in RT-qPCR experiments
*rpoD*_qPCR_R1	GTC GAA CCA CTC GCT GAT CT	Amplification of *rpoD* in RT-qPCR experiments
*fliC*_qPCR_F1	GAT GCA GAC CCA GAT CAA CG	Amplification of *fliC* in RT-qPCR experiments
*fliC*_qPCR_R1	GGA TCT GCT GCG AAA CTT CC	Amplification of *fliC* in RT-qPCR experiments
*flhD*_qPCR_F1	GTC AAC CTG TCG TAC CTC CT	Amplification of *flhD* in RT-qPCR experiments
*flhD*_qPCR_R1	CGT GAT CAT CGA AGC GGA AG	Amplification of *flhD* in RT-qPCR experiments
*motB*_qPCR_F1	CGA TGA TGG CGT TCT TCC TG	Amplification of *motB* in RT-qPCR experiments
*motB*_qPCR_R1	GCC GTT GAT GAT GCT CGA AT	Amplification of *motB* in RT-qPCR experiments
*fimA*_qPCR_F1	ATC AAC TTC ACG GGC GAG AT	Amplification of *fimA* in RT-qPCR experiments
*fimA*_qPCR_R1	GCC TGC TTG AAC TTG TTG GT	Amplification of *fimA* in RT-qPCR experiments
*bceB*_qPCR_F1	GTA TCA CAA GCT GAC GAC GC	Amplification of *bceB* in RT-qPCR experiments
*bceB*_qPCR_R1	CCG ACT TCT TGC CGT TGA TC	Amplification of *bceB* in RT-qPCR experiments
*bclA*_qPCR_F1	CAG TTC GAA TCG CTG GTG TG	Amplification of *bclA* in RT-qPCR experiments
*bclA*_qPCR_R1	CCG GAA TGA AGC GGA TGT TC	Amplification of *bclA* in RT-qPCR experiments
*bapA*_qPCR_F1	CGC AGC TTT TCA CGA TCG AT	Amplification of *bapA* in RT-qPCR experiments
*bapA*_qPCR_R1	CTG CGT GCT GAA ATC GAT CA	Amplification of *bapA* in RT-qPCR experiments
*cepR*_qPCR_F1	TAC CAG GCG CAG AAC TAC AT	Amplification of *cepR* in RT-qPCR experiments
*cepR*_qPCR_R1	CCC ACC GAC AAT CCG AAA TC	Amplification of *cepR* in RT-qPCR experiments
$hns_{1.1}$_qPCR_F1	CGA AAC TCA AGG CGC AGA TC	Amplification of $hns_{1.1}$ in RT-qPCR experiments
$hns_{1.1}$_qPCR_R1	GGC AAT CAT TCG CTG GAC AT	Amplification of $hns_{1.1}$ in RT-qPCR experiments
$hns_{1.2}$_qPCR_F1	AGT GCT GAA TGA CGT GAA GC	Amplification of $hns_{1.2}$ in RT-qPCR experiments
$hns_{1.2}$_qPCR_R1	TTC CGG ATC GCG GTA TTT TG	Amplification of $hns_{1.2}$ in RT-qPCR experiments
hns_2_qPCR_F1	ATA CGA CAT TAC GCC GGA AG	Amplification of hns_2 in RT-qPCR experiments
hns_2_qPCR_R1	CTT CGG GTT CAG GTA CTT CG	Amplification of hns_2 in RT-qPCR experiments
$hns_{3.1}$_qPCR_F1	GCA GGA ACT CGA AGC ACA AC	Amplification of $hns_{3.1}$ in RT-qPCR experiments
$hns_{3.1}$_qPCR_R1	AAG CTC GAC CTG TTC CTT GA	Amplification of $hns_{3.1}$ in RT-qPCR experiments
$hns_{3.2}$_qPCR_F1	CTG AAA GCT CAA ATG GAC GC	Amplification of $hns_{3.2}$ in RT-qPCR experiments
$hns_{3.2}$_qPCR_R1	CGT ATT CCG CGA CTT TGG TG	Amplification of $hns_{3.2}$ in RT-qPCR experiments
